# Transcriptomic insights into adenoid cystic carcinoma via RNA sequencing

**DOI:** 10.3389/fgene.2023.1144945

**Published:** 2023-04-21

**Authors:** Yu-Fang Tang, Pu-Gen An, Bao-Xin Gu, Shu Yi, Xiao Hu, Wen-Jie Wu, Jie Zhang

**Affiliations:** ^1^ Department of Oral and Maxillofacial Surgery, Peking University School and Hospital of Stomatology, Beijing, China; ^2^ National Center of Stomatology and National Clinical Research Center for Oral Diseases, Beijing, China; ^3^ Central Laboratory, Peking University School and Hospital of Stomatology, Beijing, China; ^4^ Department of Stomatology, Xinqiao Hospital (the Second Affiliated Hospital), Army Medical University, Chongqing, China

**Keywords:** adenoid cystic carcinoma (ACC), RNA sequencing, gene fusions, cancer germline antigens, mRNA

## Abstract

**Background:** The aim of this study was to investigate the underlying mechanisms of adenoid cystic carcinoma (ACC) at the transcriptome level.

**Materials and methods:** We obtained paired tumor and normal salivary gland tissues from 15 ACC patients, which were prepared for RNA sequencing.

**Results:** Gene enrichment analysis revealed that the upregulated pathways were mainly involved in axonogenesis, and the downregulated pathways were mainly related to leukocyte migration, the adaptive immune response, lymphocyte-mediated immunity, and the humoral immune response. T-cells, B-cells and NK cells showed low infiltration in ACC tissues. In addition to the gene fusions MYB-NFIB and MYBL1-NFIB, a new gene fusion, TVP23C-CDRT4, was also detected in 3 ACC tissues. PRAME was significantly upregulated in ACC tissues, while antigen-presenting human leukocyte antigen (HLA) genes were downregulated.

**Conclusion:** We found a new gene fusion, TVP23C-CDRT4, that was highly expressed in ACC. PRAME may be an attractive target for ACC immunotherapy.

## Introduction

Adenoid cystic carcinoma (ACC) is a rare and aggressive malignancy, representing 1% of head and neck cancers (Sung et al., 2021). ACC is characterized by slow local growth, a high incidence of perineural invasion (PNI), infrequent regional metastases, frequent development of local recurrences and mostly slowly progressive and relatively indolent distant metastases (Szanto et al., 1984; Tang et al., 2010). One study suggested that the overall 5-year survival rate was 68%–90%, while the 10-year and 15-year survival rates were 52% and 28%, respectively, mainly due to PNI, local recurrence and distant metastasis (DM) (Atallah et al., 2020). DM is a common malignant feature of ACC, with an incidence rate of 16.1%–72.7%. The most common site is the lung. The overall 5-year, 10-year and 20-year survival rates of patients without DM were 85.6%, 67.4%, and 50.4%, and those of patients with DM were 69.1%, 45.7%, and 14.3%, respectively (Gao et al., 2013; Jang et al., 2017; Fang et al., 2022). The incidence of PNI in ACC was 48%–82%, and PNI was an independent prognostic factor negatively correlated with prognosis. The 5-year, 10-year and 15-year disease-specific survival (DSS) rates of patients with ACC of the head and neck and with PNI were 62%, 53%, and 27%, respectively, while the DSS rates of patients without PNI were as high as 90% (Amit et al., 2015; de Morais et al., 2021). The mainstay treatment for ACC is surgical resection in combination with adjuvant radiotherapy (Sood et al., 2016). For patients with recurrent, unresectable, or metastatic ACC, chemotherapy has limited efficacy (Papaspyrou et al., 2011).

Since its first appearance in 2005 (Margulies et al., 2005), high-throughput sequencing has become a useful approach for understanding biological processes at the molecular level and conducting detailed research to elucidate the genome and transcriptome. As an essential part of high-throughput sequencing, RNA sequencing (RNA-seq) has become a powerful tool for comprehensive characterization of the whole transcriptome at the gene and exon levels, with a unique ability to identify gene alterations, gene fusions, novel splicing variants, and transcripts with high resolution and efficiency (Morozova and Marra, 2008; Wang et al., 2009; Meyerson et al., 2010; Ren et al., 2012). RNA-seq studies have made great contributions in various fields, especially in the field of cancer research, including studies on differential gene expression analysis, cancer biomarkers, cancer heterogeneity and evolution, cancer drug resistance, the cancer microenvironment and immunotherapy, and neoantigens (Hong et al., 2020). Ferraroto and colleagues revealed two molecular subtypes: ACC-I (37%) and ACC-II (63%). MYC signaling drives ACC-I via NOTCH mutations or direct amplification, and the upregulation of TP63 and receptor tyrosine kinase represents a less aggressive clinical course of ACC-II (Ferrarotto et al., 2021). Through transcriptome sequencing, Ross and co-workers found that compared with the more aggressive tumor type salivary adenocarcinoma, ACC had a significantly lower *TP53* mutation rate and tumor mutation burden (Ross et al., 2017).

However, because of the low incidence rate of ACC, only a limited number of scholars have studied ACC based on omics data. In this study, we explored 15 ACC tissues and their matched normal salivary gland (NSG) tissues through RNA-seq, attempting to discover novel markers in ACC.

## Materials and methods

We declare that all methods were carried out in accordance with the World Medical Association Declaration of Helsinki. The study was approved by the Ethics Committee and was conducted under the guidance of international ethical standards (IRB number: PKUSSIRB-202165081). Written informed consent documents were obtained from all of the patients in our study.

### Sample collection

A total of 15 pairs of ACC tumor and normal salivary gland (NSG) tissues were collected from January 2020 to March 2021. The inclusion criteria were as follows: 1. The patients with primary tumors had not received preoperative chemotherapy, radiotherapy, or any other anticancer treatment prior to surgery. 2. The patients received surgical treatment for primary tumors in our hospital. 3. The tumors were confirmed as ACC by two pathologists from the Department of Diagnostic Pathology in our hospital. The exclusion criteria were as follows: 1. Patients who declined to participate in the study; 2. Recurrent or metastatic ACC samples. NSG tissues were collected from the submandibular glands of patients with primary ACC undergoing neck dissection. Tissue specimens were freshly frozen immediately after surgery and stored at −80°C.

### RNA extraction and qualification

RNA was extracted from tissues using TRIzol (Invitrogen, California, United States) according to the manufacturer’s instructions. Fresh samples were ground to fine powder in liquid nitrogen, transferred to a tube, mixed with 1 mL of TRIzol, and then kept at room temperature for 10 min. Then, 200 μL of chloroform was added, and the sample was centrifuged (12,000 rpm, 10 min) at 4°C. An equal volume of phenol:chloroform (v:v 25:24) was added to the supernatant and centrifuged for 10 min at 12,000 rpm and 4°C. The supernatant was added to an equal volume of chloroform and centrifuged for 10 min at 12,000 rpm and 4°C. An equal volume of isopropyl alcohol was added to the supernatant; the sample was kept at −20°C for 1 h, and then centrifuged for 10 min at 12,000 rpm at 4°C; the supernatant was discarded. The pellet was washed with 1 mL of 75% ethanol and centrifuged (X 8000 rpm, 5 min) at 4°C, and the supernatant was discarded. The residual ethanol was removed by pipetting and dried under a vacuum. Finally, 50 μl of RNase-free water was added to dissolve the RNA (Wei et al., 2022). RNA degradation and contamination were monitored on 1% agarose gels. RNA purity was checked using a NanoPhotometer^®^ spectrophotometer (IMPLEN, CA, United States). RNA integrity was assessed using the RNA Nano 6000 Assay Kit of the Bioanalyzer 2100 system (Agilent Technologies, CA, United States).

### Library preparation for transcriptome sequencing

A total of 1 µg RNA per sample was used as input material for the RNA sample preparations. Sequencing libraries were generated using the NEBNext^®^ UltraTM RNA Library Prep Kit for Illumina^®^ (NEB, United States) following the manufacturer’s recommendations, and index codes were added to attribute sequences to each sample. Briefly, mRNA was purified from total RNA using poly-T oligo-attached magnetic beads. Fragmentation was carried out using divalent cations under elevated temperature in NEBNext First Strand Synthesis Reaction Buffer (5X). First-strand cDNA was synthesized using random hexamer primers and M-MuLV Reverse Transcriptase (RNase H). Second-strand cDNA synthesis was subsequently performed using DNA polymerase I and RNase H. The remaining overhangs were converted into blunt ends via exonuclease/polymerase activities. After adenylation of the 3′ ends of DNA fragments, NEBNext adaptors with hairpin loop structures were ligated in preparation for hybridization (Zhu et al., 2018). To preferentially select cDNA fragments 250–300 bp in length, the library fragments were purified with the AMPure XP system (Beckman Coulter, Beverly, United States). Then, 3 µL USER Enzyme (NEB, United States) was incubated with size-selected, adaptor-ligated cDNA at 37°C for 15 min followed by 5 min at 95°C before PCR. Then, PCR was performed with Phusion High-Fidelity DNA polymerase, universal PCR primers and Index (X) Primer (Zhu et al., 2018). Finally, PCR products were purified (AMPure XP system), and library quality was assessed on the Agilent Bioanalyzer 2100 system (Wei et al., 2022).

### Clustering and sequencing

Clustering of the index-coded samples was performed on a cBot Cluster Generation System using TruSeq PE Cluster Kit v3-cBot-HS (Illumina) according to the manufacturer’s instructions. After cluster generation, the library preparations were sequenced on an Illumina NovaSeq platform, and 150 bp paired-end reads were generated (Wei et al., 2022).

### Analysis of RNA-seq data

Quality control analyses of raw reads were first performed by FastQC and MultiQC, followed by read trimming by FastP. Filtered data were then mapped to GENCODE v37. The reads of the transcripts were further aligned and quantified against GENCODE human transcriptome v37 using Salmon (v1.4.0), imported and summarized in R by tximeta. The median number of reads per sample was 20.4 million. DESeq2 (Yu et al., 2012; Love et al., 2014) was then used to perform exploratory analysis and differential expression analysis. PCA suggested that two normal tissue samples were grouped with tumor tissues, suggesting that they were outliers of the normal tissues. These two normal tissue samples were excluded from the following analysis. *p* values calculated by Wald statistics were corrected by Benjamini‒Hochberg methods. Adjusted *p* values lower than 0.05 were considered significant. Functional enrichment of differentially expressed genes (DEGs) using gene sets from Gene Ontology (GO) and Kyoto Encyclopedia of Genes and Genomes (KEGG) and MSigDB hallmark gene sets was performed with clusterProfiler. For gene fusion analysis, prefiltered reads were first aligned against the genome reference from CTAT_GENOME_LIB (GRCh38_gencode_v37) by STAR. Candidate fusion transcripts were identified by STAR-Fusion and further visualized by visualization tools provided by Arriba (Haas et al., 2019; Uhrig et al., 2021). Identification and analysis of isoform switches were performed by IsoformSwitchAnalyzeR using default settings (Vitting-Seerup and Sandelin, 2019).

### Immunohistochemistry

The tissue samples for immunohistochemistry were collected from the tumors and the submandibular glands of the patients with primary ACC of head and neck undergoing neck dissection. Twelve pairs of paraffin-embedded ACC and NSG tissues were sectioned coronally to generate 3 μm slices. The sections were incubated with the following primary antibodies as indicated: CD3 (Gene Tech, GA045207) at a 1:20 dilution ratio; CD4 (Gene Tech, GT219107) at a 1:20 dilution ratio; CD8 (Gene Tech, GT211207) at a 1:20 dilution ratio and CD19 (Gene Tech, GT212802) at a 1:50 dilution ratio. The samples were stained with horseradish peroxidase-labeled secondary antibody (Bond Polymer Refine Detection, Leica; RS9800) and hematoxylin. The slides were photographed using a Leica microscope. IHC images were analyzed by 2 pathologists. They randomly selected five images of the peritumoral and intratumoral regions of ACC and NSG under ×400 magnification. Then, Image-Pro Plus 6.0 (Media Cybernetics, Rockville, MD, United States) was used to automatically count the number of positive cells and manually remove false-positive cells. The cell density = the total number of positive cells in 5 pictures/0.125 mm^2^.

## Results

### Clinicopathological characteristics

**FIGURE 1:**
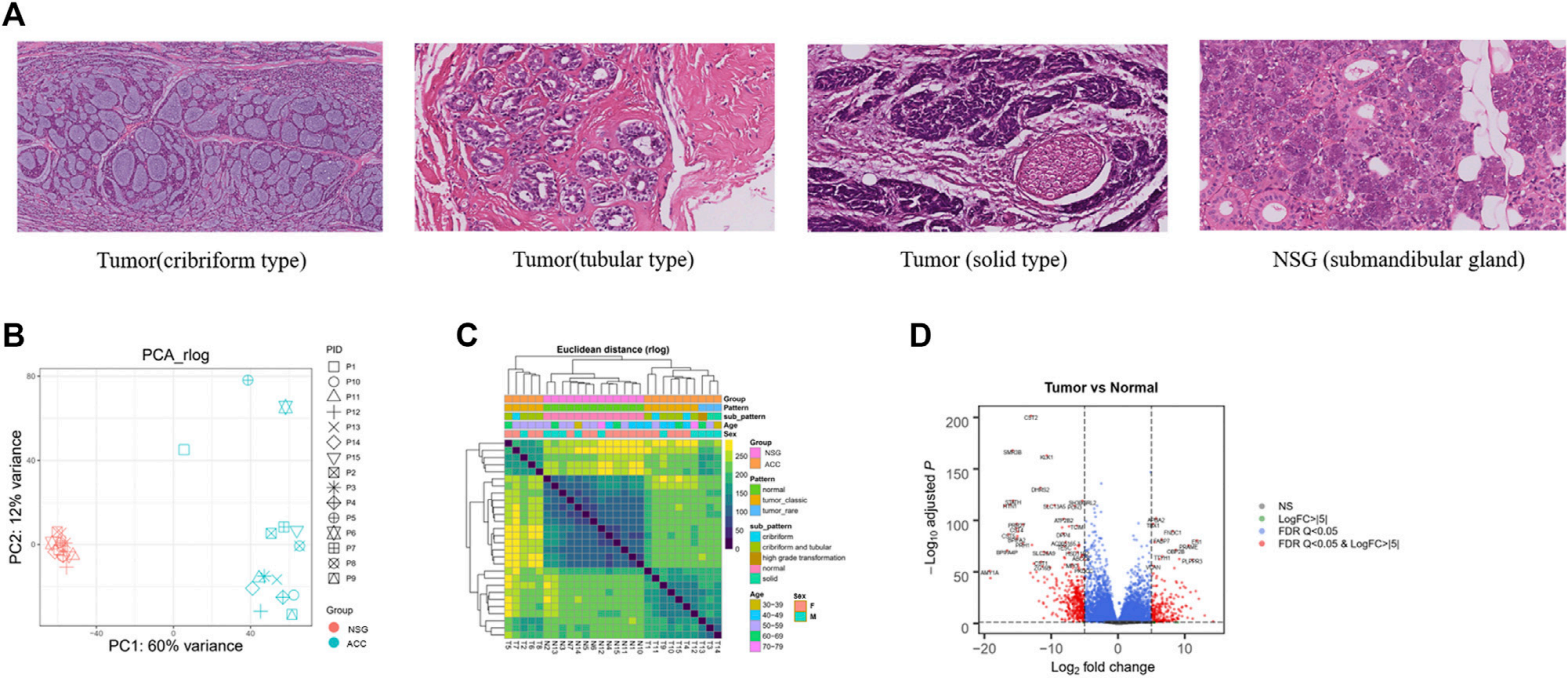
H&E and DEGs between tumor and NSG tissues. **(A)** The histopathological features of the ACCs and NSG used in this study (H&E, 40x). **(B)** PCA plots showing clustering of 28 samples (13 NSG and 15 ACCs). **(C)** Sample distances showing clustering of 28 samples (13 NSG and 15 ACCs) (tumor_classic: ACC with a pathological type of cribriform-tubular and cribriform; tumor_rare: ACC with a pathological type of sold and high-grade transformation). **(D)** Volcano plot of the DEGs. The red (top) and blue (down) sites reflect the differentially expressed RNAs with log fold-change>│5│and PDR Q < 0.05.

To gain insight into the molecular pathogenesis of ACC, we collected 15 ACC tissues and 15 paired NSG tissues. The clinicopathological information of patients with ACC is shown in Table 1, including age, sex, tumor location, pathological type, PNI status, and TNM classification. Figure 1A illustrates the histopathological features of ACC. The tumor tissue presents cribriform or tubular changes.

**TABLE 1:** The clinicopathological characteristics of the 15 patients.

Variable	No. of cases
Age	
Median	56
Range	42–73
Sex	
Female	9
Male	6
Location	
Maxillary sinus	4
Base of tongue	1
Palate	8
Sublingual gland	2
Pathological type	
Cribriform-tubular	9
Solid	2
Cribriform	3
High-Grade transformation	1
Perineural invasion	
Positive	5
Negative	10
T stage	
T3	5
T4	10
N stage	
N0	14
N2	1
M stage	
M0	13
M1	2

### Exploratory analysis and DEG analysis of ACC samples

After RNA-seq and preliminary filtration, a total of 15 ACC and 13 NSG samples were subjected to downstream analysis. NSG tissues from two cases failed the quality inspection. DESeq2 (Yu et al., 2012; Love et al., 2014) was then used to perform exploratory analysis and differential expression analysis. PCA and sample distances were used to assess the differences between ACC tissues and NSG tissues. PCA plots showed that the first principle component (PC1) accounted for 60% of the overall variance of the data, and the second principle component (PC2) accounted for 12% (Figure 1B), suggesting an obvious difference between the two groups. The heatmap of intersample correlation is shown in Figure 1C. The results showed that tumor and normal tissues were clustered separately, indicating distinct changes in the transcriptome in tumor samples.

The DEG analysis identified a total of 6967 DEGs (3520 upregulated and 3447 downregulated) in 15 ACC tissues compared with 13 NSG tissues (*p* < 0.05 and log2-fold-change>1). The top 20 DEGs are shown in Table 2. The most upregulated genes in the tumor group included EN1, PRAME, FNDC1, APBA2, TBX1, FABP7, CBP2B, and TTYH1, while CST2, SMR3B, KLK1, CTBS, THN1, and STATH were significantly decreased, as shown in the volcano plot (Figure 1D).

**TABLE 2:** Top 20 up and downregulated DE-mRNAs.

gene_name	log2FoldChange	Padj	Sig
COL27A1	4.905059189	1.25E-147	up
APBA2	5.695475595	8.12E-103	up
TUBA1A	3.071934334	5.56E-98	up
TBX1	5.253479631	1.80E-97	up
FNDC1	8.205559027	2.53E-90	up
PLSCR3	2.312919959	6.29E-86	up
FABP7	6.549525418	2.88E-82	up
TP53	2.431556017	3.67E-82	up
ABCC1	2.789483537	8.20E-82	up
EN1	11.81068084	2.06E-81	up
MFAP2	4.852118182	4.55E-80	up
MEX3A	5.502988401	1.08E-79	up
PRAME	10.57626928	1.76E-76	up
STMN1	4.323275057	4.82E-76	up
TRO	3.533004668	1.69E-75	up
OBP2B	8.656299902	3.33E-72	up
ITGA9	4.683738203	4.26E-69	up
TTYH1	6.612421301	6.53E-66	up
SOX4	3.716569748	4.25E-65	up
CASC15	3.099970158	1.51E-63	up
CST2	−13.02941499	3.07E-202	down
SMR3B	−15.78505533	1.02E-168	down
KLK1	−10.66554013	7.85E-164	down
CTBS	−2.505495637	1.19E-136	down
DHRS2	−11.62188281	2.85E-132	down
STATH	−15.7089987	6.94E-120	down
SH3BGRL2	−5.323275759	1.04E-119	down
HTN1	−16.16367832	3.66E-116	down
SLC13A5	−9.511121749	1.22E-115	down
SLC9A1	−3.119844922	6.99E-115	down
HTN3	−16.96742223	6.99E-115	down
PON3	−6.48883633	7.48E-115	down
FXYD2	−10.55561278	7.48E-115	down
WWC1	−3.561034857	5.02E-112	down
PDCD4	−2.542553396	5.15E-109	down
CTPS1	−3.47144039	3.20E-108	down
FUT8	−3.351754045	2.60E-104	down
ATP2B2	−8.112838869	2.43E-102	down
BLM	−4.766716988	6.01885E-100	down
PRR27	−15.16202799	1.38806E-97	down

### Functional annotation and pathway enrichment analysis

**FIGURE 2:**
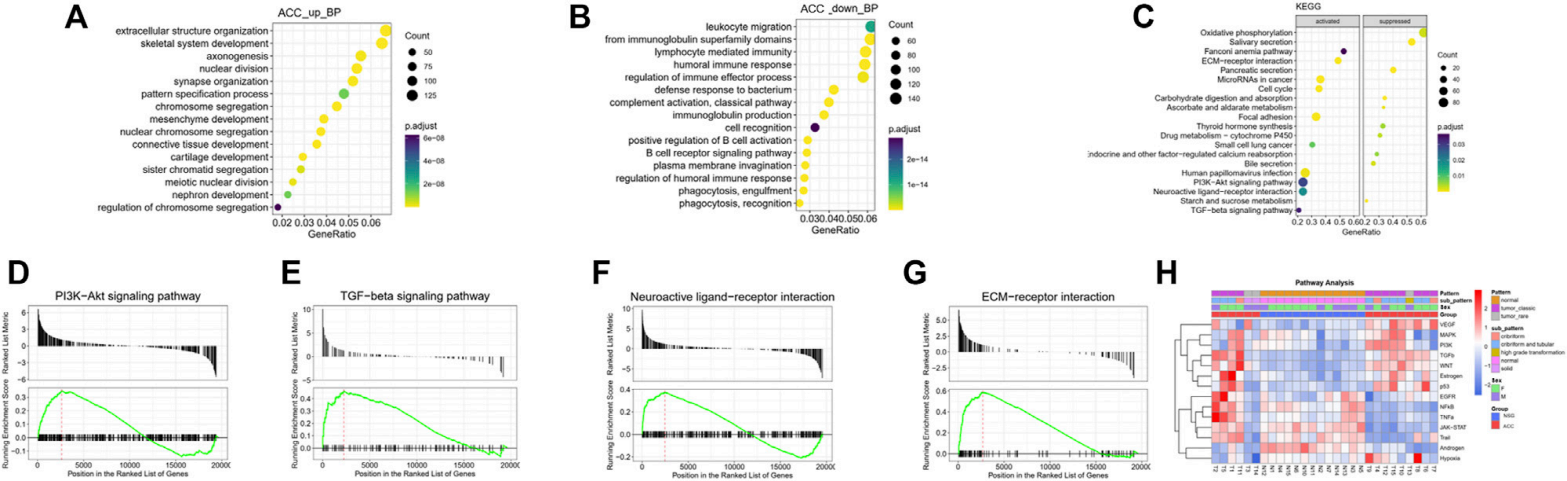
GO and KEGG analyses of the DEGs. **(A)** The top 15 upregulated GO BP terms of the DEGs were identified by GO analysis. **(B)** The top 15 downregulated GO BP terms of the DEGs were identified by GO analysis. **(C)** The top 20 enriched pathways of the DEGs, including the top 10 activated and 10 suppressed pathways, were identified by KEGG enrichment analysis. **(D–G)** GSEA plots of specific activated pathway terms, including PI3K-Akt, TGF-beta, neuroactive ligand‒receptor interaction, and ECM-receptor interaction signaling pathway. **(H)** Further signaling pathway analysis by subtype revealed that some classical pathways, the VEGF, MAPK, PI3K, TGFb, WNT, estrogen, and p53 pathways, were activated.

GO and KEGG pathway enrichment analyses were performed to further understand the biological significance of the DEGs. The significantly enriched GO biological process (BP) terms of the upregulated pathways in the tumor samples were primarily related to extracellular structure organization, skeletal system development, nuclear division, and axonogenesis (Figure 2A), indicative of changes in cell proliferation and the tumor microenvironment. The significantly enriched GO BP terms of the downregulated pathways were mainly involved in leukocyte migration, the adaptive immune response, lymphocyte-mediated immunity, and the humoral immune response, suggesting a lack of immune response in local ACC tissues (Figure 2B).

Gene set enrichment analysis (GSEA) of KEGG pathways, including both activated and suppressed pathways, was also performed (Figure 2C). In our analysis, activated pathways in the ACC groups were mainly related to the terms EMC-receptor interaction, microRNAs in cancer, focal adhesion, and neuroactive ligand‒receptor interaction, consistent with the GO BP analysis. The suppressed pathways were mainly related to oxidative phosphorylation, salivary secretion, pancreatic secretion, and several metabolic pathways, suggesting loss of normal salivary secretion function and changes in cell metabolism. The GSEA plots of specific activated pathways, including pathways related to PI3K-Akt, TGF-beta, neuroactive ligand‒receptor interactions, and ECM-receptor interactions, are also shown in Figures 2D–G.

Further signaling pathway analysis by ACC subtype revealed that some classical pathways, the VEGF, MAPK, PI3K, TGFb, WNT, estrogen, and p53 pathways, were activated (Figure 2H).

Furthermore, as seen from the signaling pathway results, most of these pathways were inactivated in solid-type ACC, suggesting that therapy in this subtype may be different from that in other pathological types (cribriform-tubular or cribriform).

### Screening of hub genes suggests dynamic changes in the immune microenvironment

**FIGURE 3:**
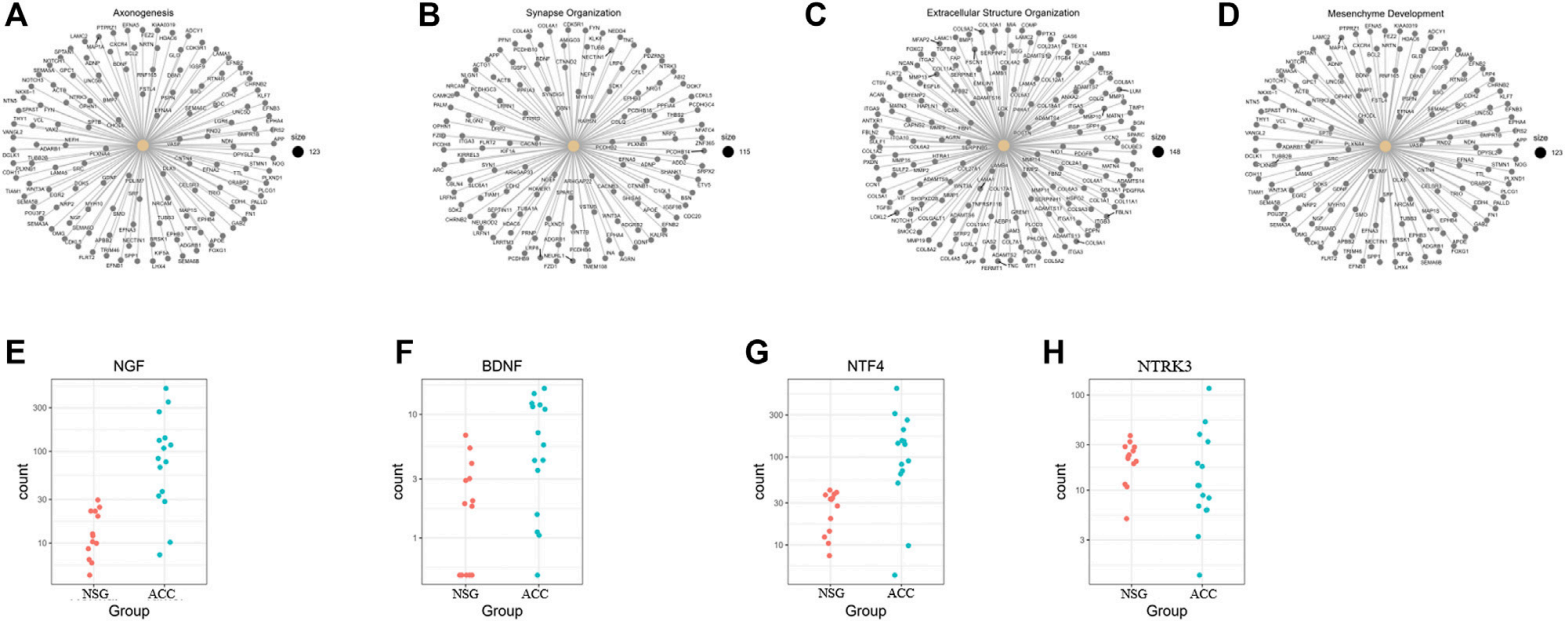
Genes related to the enriched upregulated GO BP terms. **(A–D)** Genes involved in axonogenesis, synapse organization, extracellular structure organization, and mesenchyme development. **(E–H)** NGF, BDNF, NTF4, and NTRK3 were obviously upregulated axonogenesis-related genes.

According to the GO BP enrichment analysis, genes involved in axonogenesis, synapse organization, extracellular structure organization, and mesenchyme development were enriched, as shown in Figures 3A–D. Among the axonogenesis-related upregulated genes, genes encoding neurotrophins and their receptors, including NGF, BDNF, NTF4, and NTRK3, were significantly correlated, as shown in Figures 3E–H. These data suggest a possible close relationship between ACC and PNI.

**FIGURE 4:**
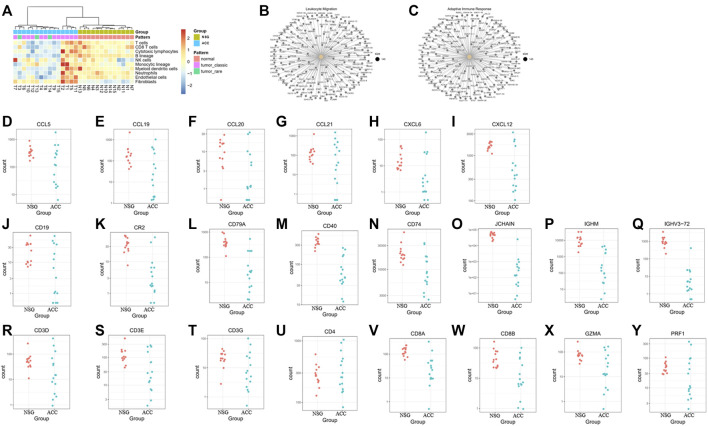
The levels of immune cells, endothelial cells, and fibroblasts in ACC tissues and downregulated DEGs in the GO BP enrichment. **(A)** The levels of CD3^+^ T-cells, CD8^+^ T-cells, cytotoxic lymphocytes, natural killer cells, B lymphocytes, monocytic lineage cells, myeloid dendritic cells, neutrophils, endothelial cells and fibroblasts in ACC. **(B–C)** Downregulated genes involved in leukocyte migration and the adaptive immune response. **(D–I)** Downregulated genes involved in leukocyte migration, including CCL5, CCL19, CCL20, CCL21, CXCL6, and CXCL12. **(J–Q)** Downregulated B-cell-related genes, including CD19, CR2, CD79A, CD40, CD74, JCHAIN, IGHM, and IGHV3-72. **(R–Y)** Downregulated T cell-related genes, including CD3E, CD3G, CD4, CD8A, CD8B, GZMA, and RPF1.

Some investigators have demonstrated that ACC exhibits low infiltration of antitumor immune cells (being a cold tumor). In our study, deconvolution of the cellular composition data was also performed. The R package MCP-counter (18) was applied to the normalized log2-transformed FPKM expression matrix to produce the absolute abundance scores for eight major immune cell types (CD3^+^ T-cells, CD8^+^ T-cells, cytotoxic lymphocytes, natural killer (NK) cells, B lymphocytes, monocytic lineage cells, myeloid dendritic cells and neutrophils), endothelial cells and fibroblasts. The deconvolution profiles were then hierarchically clustered and compared across the control and ACC groups (Figure 4A).

The downregulated genes involved in leukocyte migration and the adaptive immune response are shown in Figures 4B, C. The most significantly downregulated leukocyte migration-related genes included CCL5, CCL19, CCL20, CCL21, CXCL6, and CXCL12 (Figures 4D–I). The expression of B-cell-related and immunoglobulin genes, including CD19, CR2, CD79A, CD40, CD74, JCHAIN, IGHM, and IGHV3-72, was also downregulated (Figure 4J–Q). Expression of T-cell-related genes, mainly cytotoxic T and NK cell-related genes (including CD3D, CD3E, CD3G, CD4, CD8A, CD8B, GZMA, and RPF1), was also downregulated (Figure 4R–Y).

**FIGURE 5:**
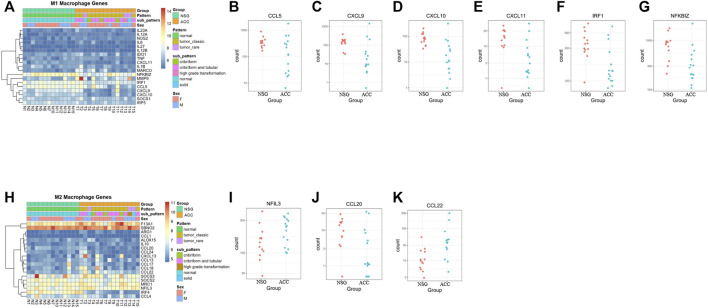
The levels of M1/M2 macrophages. **(A–G)** Expression of M1 macrophage-related genes. **(H–K)** Expression of M2 macrophage-related genes.

Regarding M1/M2 macrophages, most M1 macrophage-related genes were downregulated, whereas 4 M2 macrophage-related genes were upregulated and 1 was downregulated, suggesting that overall inflammation was downregulated (Figures 5A–K).

**FIGURE 6:**
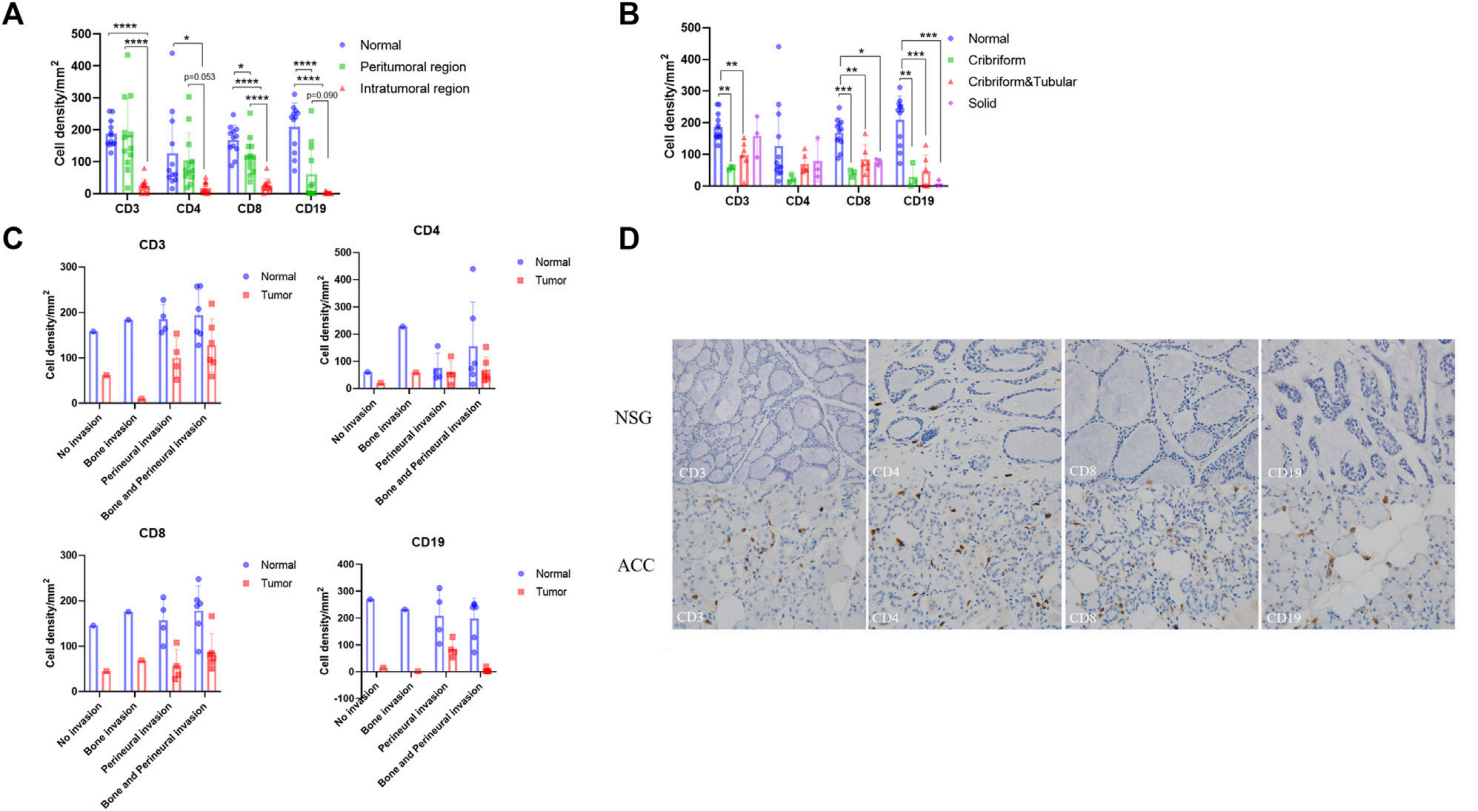
Immunohistochemistry of T cells and B cells in ACC tissues and NSG tissues. Brownish yellow-positive granules were specifically located on the cell membrane. **(A)** The expression of CD3, CD4, CD8, and CD19 in peritumoral and intratumoral regions. **(B)** The expression of CD3, CD4, CD8, and CD19 in tumor tissues of three pathological types. **(C)** The expression of CD3, CD4, CD8, and CD19 in tumor tissues of different invasion types. **(D)** The expression of CD3, CD4, CD8, and CD19 in ACC and NSG tissues (immunohistochemistry stain; ×400).

To confirm the changes in immune infiltration in ACC tissues, we further performed immunohistochemistry staining for CD3, CD4, CD8, and CD19 in the ACC tissues. We used the staining intensity of cells to assess the immune infiltration level of these markers and analyzed their differences by one-way ANOVA-Tukey’s multiple comparisons test. The Supplementary Materials show these statistical data. We first compared the expression differences of these markers in the normal and peritumoral and intratumoral regions (Figure 6A). Compared with that in normal tissues, the expression of CD3, CD4, CD8, and CD19 in ACC tissues decreased, and the expression in the intratumoral region decreased more significantly than that in the peritumoral region. In addition, the expression levels of these markers were obviously different among the three pathological types of ACC (Figure 6B). Lower expression levels of CD3, CD4, and CD8 were observed in cribriform ACCs, while the expression level of CD19 was the lowest in solid ACCs. Finally, there was a significant difference in the expression level of these markers in different tumor invasion types (Figure 6C). The lowest expression level of CD3 was observed in the bone-invasive type, and CD4 and CD8 were observed in the non-invasive type. The expression level of CD19 was generally low in all types. Consistent with the above RNA-seq results, the immunohistochemistry results showed decreased infiltration of T-cells and B-cells in the tumor microenvironment compared to the normal tissues (Figure 6D).

### Detection of gene fusions

**FIGURE 7:**
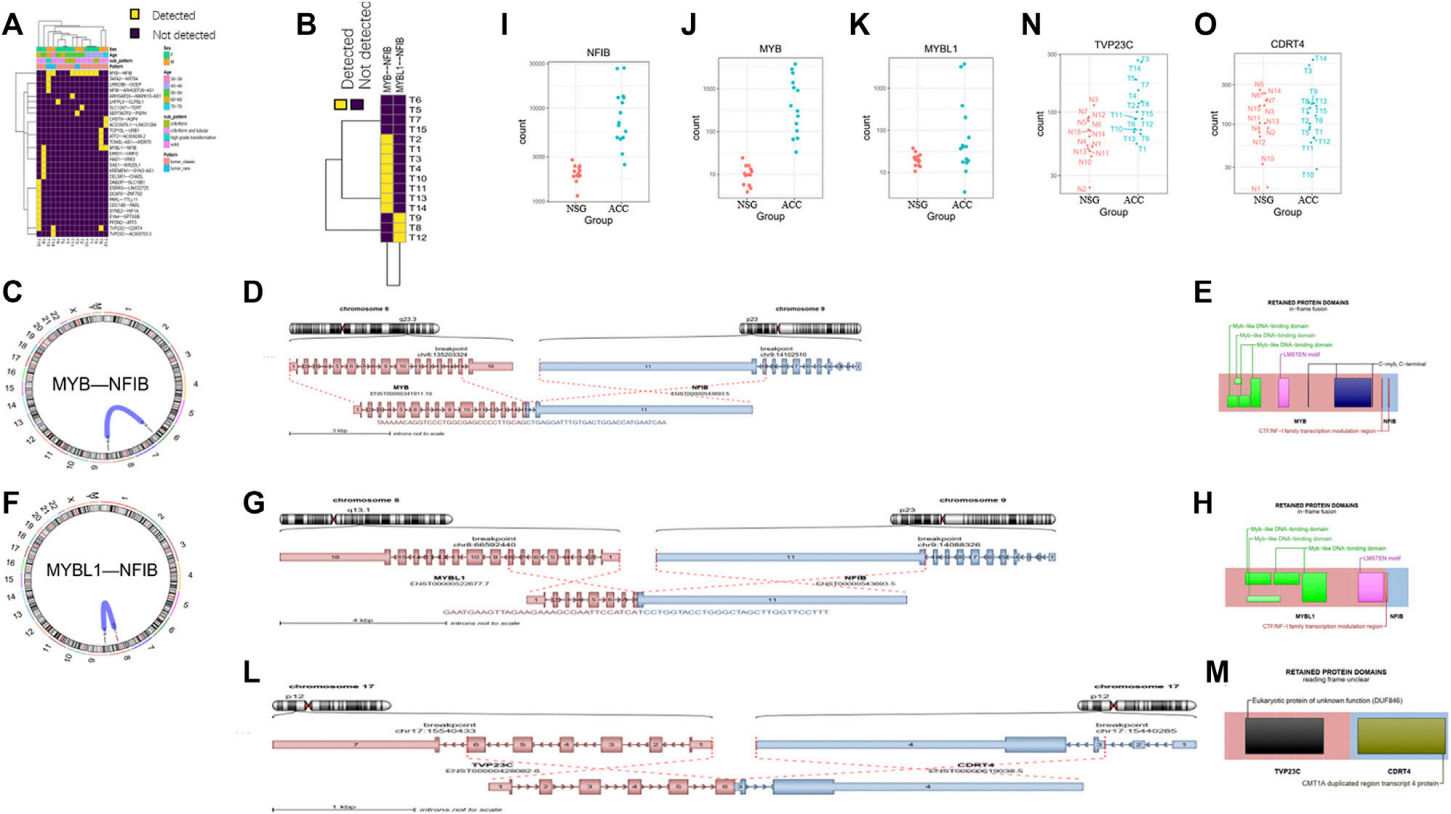
Detection of gene fusions. **(A)** Twenty-nine gene fusions were detected in ACC tissues. **(B)** Eleven NFIB-related gene fusions were detected in 15 samples. **(C–E)** The fusion gene of the transcription factors MYB and NFIB consists of different regions from chromosomes 6 and 9. **(F–H)** The fusion gene of the transcription factors MYBL1 and NFIB consists of different regions from chromosomes 8 and 9. **(I–K)** The gene expression of NFIB, MYB and MYBL1 in tumor samples. **(L–M)** TVP23C-CDRT4 gene fusions including different regions of chromosome 17. **(N–O)** TVP23C and CDRT4 were upregulated in the T3 and T14 tumor samples.

In addition to DEG analysis, we also performed analysis of gene fusions. In our 15 ACC samples, a total of 29 possible gene fusions were detected (Figure 7A). The breakpoints of different types of gene fusions are shown in Table 3. Among them, 11 NFIB-related gene fusions were detected in 15 samples (Figure 7B), and there were different subtypes of fusions (Table 4). The gene fusions involving the transcription factors MYB and NFIB consisted of different regions in chromosomes 6 and 9, as shown in Figures 7C–E, while parts of chromosomes 8 and 9 fused to form the fusion gene of the transcription factors MYBL1 and NFIB (Figures 7F–H). The fusion of genes may change gene functions via different regulatory mechanisms. The gene expression of NFIB and MYB was significantly upregulated in most samples, while that of MYBL1 was also upregulated in 5 samples (Figures 7I–K). Moreover, the gene fusion TVP23C-CDRT4 was also detected in three ACC tissues, and different types of fusions were detected, as shown in Table 4. TVP23C-CDRT4 gene fusions include different regions of chromosome 17, and the fusion structures are shown in Figures 7L, M. In addition, the expression of TVP23C and CDRT4 was significantly upregulated in 2 ACC samples, and the pathological subtype of these 2 samples was solid type (Figures 7N, O).

**TABLE 3:** Gene fusions in adenoid cystic carcinoma.

Sample	FusionName	LeftBreakpoint	RightBreakpoint
T1	MYB--NFIB	chr6:135194460:+	chr9:14088326:−
	MYB--NFIB	chr6:135194451:+	chr9:14088326:
T2	SLC12A7--TERT	chr5:1073633:−	chr5:1272280:−
	MYB--NFIB	chr6:135200415:+	chr9:14088326:−
T3	MYB--NFIB	chr6:135196002:+	chr9:14102510:−
	MYB--NFIB	chr6:135196002:+	chr9:14088326:−
	SEPTIN7P2--PSPH	chr7:45768660:−	chr7:56021231:−
T4	MYB--NFIB	chr6:135203324:+	chr9:14102510:−
	MYB--NFIB	chr6:135203324:+	chr9:14088326:−
T5	ND		
T6	LHFPL5--CLPSL1	chr6:35797459:+	chr6:35786998:+
T7	ND		
T8	MYBL1--NFIB	chr8:66592440:−	chr9:14088326:−
	KREMEN1--SYN3-AS1	chr22:29073227:+	chr22:32583877:+
	CELSR1--CHADL	chr22:46533627:−	chr22:41229730:−
	SAE1--KIR2DL1	chr19:47131028:+	chr19:54775165:+
	HAS1--VRK3	chr19:51723925:−	chr19:50016163:−
	EMID1--GMFG	chr22:29206139:+	chr19:39333126:−
T9	MYBL1--NFIB	chr8:66592440:−	chr9:14088326:−
	ATF2--AC009299.2	chr2:175093061:−	chr2:161223483:−
	TVP23C--CDRT4	chr17:15540433:−	chr17:15440285:−
	TONSL-AS1--WDR70	chr8:144437746:+	chr5:37516514:+
	TCP10L--URB1	chr21:32567941:−	chr21:32385684:−
T10	MYB--NFIB	chr6:135196002:+	chr9:14102510:−
T11	MYB--NFIB	chr6:135203324:+	chr9:14102510:−
	ARHGAP24--MAPK10-AS1	chr4:85995657:+	chr4:86119984:+
T12	MYBL1--NFIB	chr8:66592440:	chr9:14088326:
	CHST9--AQP4	chr18:27024116:−	chr18:26862596:−
	AC233976.1--LINC01284	chrX:51356944:−	chrX:51171871:
T13	LRRC8B--OOEP	chr1:89525022:+	chr6:73369385:−
	MYB--NFIB	chr6:135194460:+	chr9:14102510:−
	NFIB--ARHGEF26-AS1	chr9:14120440:−	chr3:154090331:−
	MYB--NFIB	chr6:135194451:+	chr9:14102510:
	LRRC8B--OOEP	chr1:89549971:+	chr6:73369385:−
	TAFA2--KRT84	chr12:62191259:−	chr12:52383798:−
	TAFA2--KRT84	chr12:62191259:−	chr12:52386990:−
	LRRC8B--OOEP	chr1:89549971:+	chr6:73394488:−
T14	MYB--NFIB	chr6:135203324:+	chr9:14102510:−
	MYB--NFIB	chr6:135203324:+	chr9:14088326:−
	MYB--NFIB	chr6:135203324:+	chr9:14113081:−
	TVP23C--CDRT4	chr17:15540433:−	chr17:15440285:−
	TVP23C--CDRT4	chr17:15545785:−	chr17:15438200:−
	TVP23C--AC005703.3	chr17:15540433:−	chr17:15416233:−
T15	EYA4--SPTSSB	chr6:133274813:+	chr3:161359894:−
	PFDN2--ATF3	chr1:161117952:−	chr1:212615018:+
	SYNE2--HIF1A	chr14:63853143:+	chr14:61720382:+
	CDC14B--PARL	chr9:96619219:−	chr3:183866765:−
	PARL--TTLL11	chr3:183884722:−	chr9:122039368:−
	DCAF6--ZNF75D	chr1:167937008:+	chrX:135296039:−
	CDC14B--PARL	chr9:96619219:−	chr3:183844326:−
	ESRRG--LINC02725	chr1:216564219:−	chr11:128180460:+
	DAB2IP--SLC18B1	chr9:121598626:+	chr6:132774313:−
	TVP23C--CDRT4	chr17:15540433:−	chr17:15440285:−

**TABLE 4:** Different types of gene fusions.

		LeftBreakpoint	RightBreakpoint
MYB—NFIB	T1	chr6:135194460:+	chr9:14088326:−
		chr6:135194451:+	chr9:14088326:
	T2	chr6:135200415:+	chr9:14088326:−
	T3	chr6:135196002:+	chr9:14102510:−
		chr6:135196002:+	chr9:14088326:−
	T4	chr6:135203324:+	chr9:14102510:−
		chr6:135203324:+	chr9:14088326:−
	T10	chr6:135196002:+	chr9:14102510:−
	T11	chr6:135203324:+	chr9:14102510:−
	T13	chr6:135194460:+	chr9:14102510:−
		chr6:135194451:+	chr9:14102510:−
	T14	chr6:135203324:+	chr9:14102510:−
		chr6:135203324:+	chr9:14088326:−
		chr6:135203324:+	chr9:14113081:−
MYBL1--NFIB	T8	chr8:66592440:−	chr9:14088326:−
	T9	chr8:66592440:−	chr9:14088326:−
	T12	chr8:66592440:−	chr9:14088326:−
TVP23C—CDRT4	T9	chr17:15540433:−	chr17:15440285:−
	T14	chr17:15540433:−	chr17:15440285:−
		chr17:15545785:−	chr17:15438200:−
	T15	chr17:15540433:−	chr17:15440285:−

### Expression of cancer germline antigens

**FIGURE 8:**
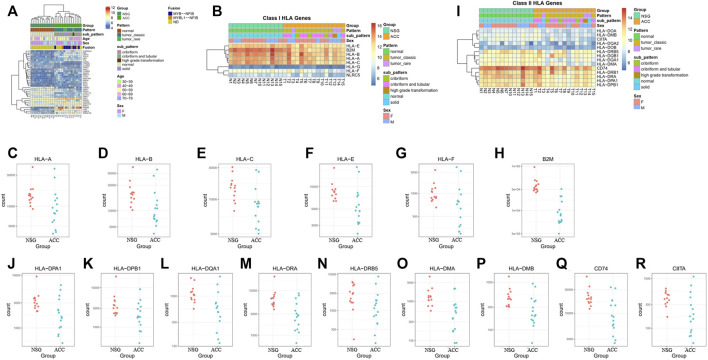
Expression of cancer germline antigens. **(A)** Expression of 35 tumor antigens in ACC. **(B–H)** The expression of class I HLA molecules (HLA-A, HLA-B, HLA-C, HLA-D, HLA-E, HLA-F, and B2M) was downregulated in some patients. **(I–R)** The expression of class II HLA molecules (HLA-DPA1, HLA-DPB1, HLA-DQA1, HLA-DRA, HLA-DRB5, HLA-DMA, HLA-DMB, and CIITA) was also downregulated in some patients.

Cancer germline antigens play an important role in the occurrence and progression of tumors. To find novel markers, we further screened cancer germline antigens. The top 35 antigens expressed differently in the ACC tissues vs. NSG tissues were detected. Among them, PRAME was the most significantly upregulated in all ACC samples. The results of the analysis of cancer germline antigen expression in different patients are shown in Figure 8A.

Human leukocyte antigen (HLA) molecules are required to present antigens to immune cells to elicit an efficient immune response. The expression of class I HLA molecules, which are responsible for antigen presentation to tumor-killing T-cells, including HLA-A, HLA-B, HLA-C, HLA-E, HLA-F, and B2M, was downregulated in 7, 8, 5, 10, 5, and 14 patients, respectively (Figures 8B–H). The expression of class II HLA molecules, which are responsible for antigen presentation to helper T cells, including HLA-DPA1, HLA-DPB1, HLA-DQA1, HLA-DRA, HLA-DRB5, HLA-DMA, HLA-DMB, CD74, and CIITA, was also downregulated in 8, 5, 5, 10, 6, 7, 9, 9, and 7 patients, respectively (Figures 8I–R).

### Different isoform analysis of mRNAs

**FIGURE 9:**
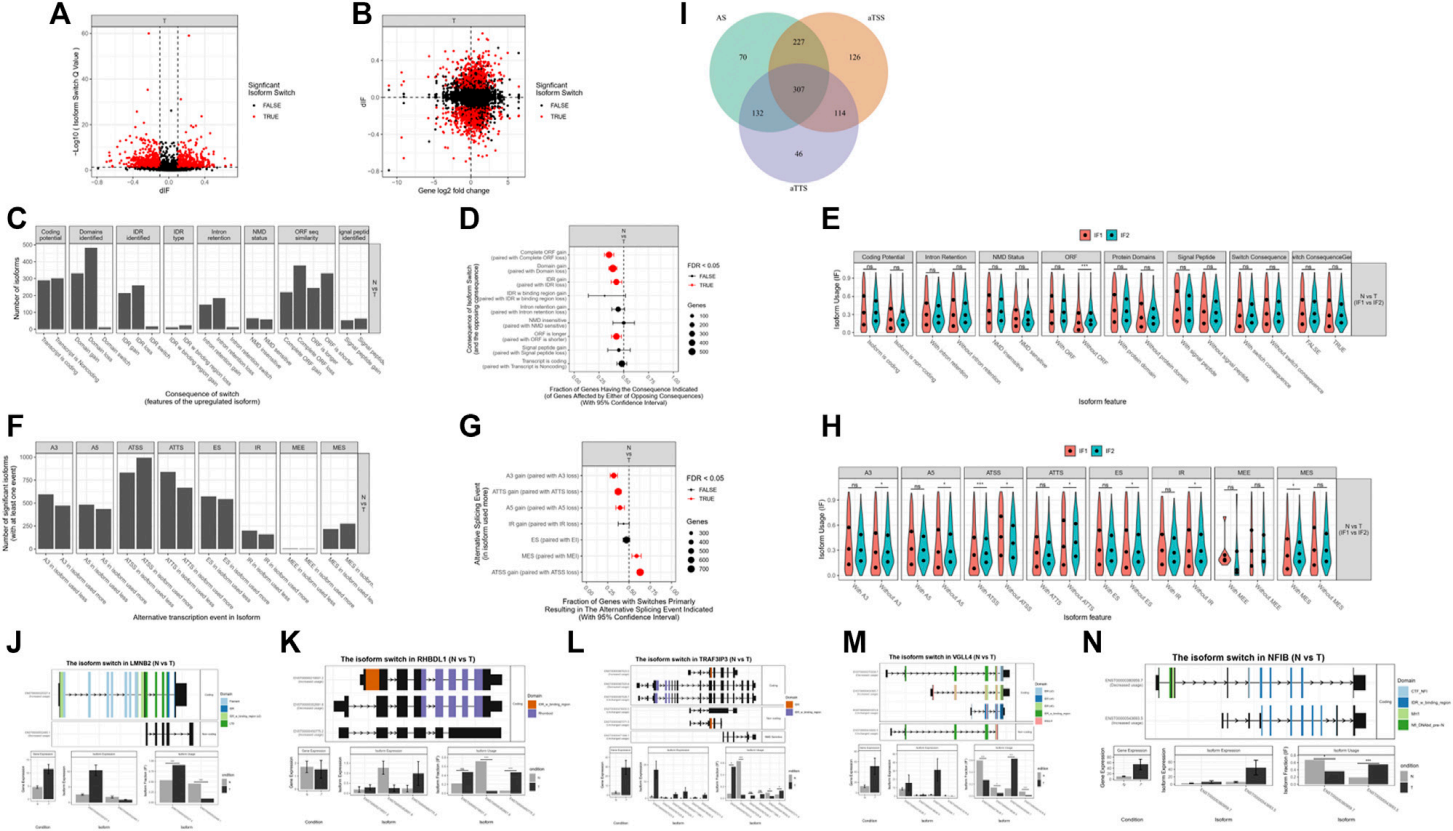
mRNAs isoform analysis. **(A)** A total of 1690 isoforms were identified for all significant genes. **(B)** A total of 1336 isoforms were identified with potential functional consequences. **(C–E)** Genome-wide analysis of isoform switches and their predicted consequences. **(F–H)** Genome-wide analysis of alternative splicing and changes. **(I)** Analysis of the biological mechanisms behind isoform switching. **(J–N)** Top genes associated with isoform switching.

In addition to differential expression of genes, mRNA isoform switching may also lead to functional changes in genes, even without significant changes in total gene expression. Therefore, we also performed isoform switching analysis in our samples. We identified 1,690 isoformEs for all significant genes: 1,318 pairs of isoforms had significantly changed usage in opposite directions, and 1,162 genes had significantly differential transcript usage. Among them, 1,336 isoforms were identified with potential functional consequences: 1,063 pairs of isoforms had significantly changed usage in opposite directions, and 922 genes had significantly differential transcript usage (Figures 9A, B).

Genome-wide analysis of isoform switches revealed that, compared to normal tissues, ACCs had a significantly higher fraction of switches resulting in complete open reading frame (ORF) loss, domain loss, intrinsically disordered region (IDR) loss, and shortened ORFs (Figures 9C–E). In the genome-wide analysis of alternative splicing and changes, ACCs had a significantly higher fraction of switches, resulting in loss of alternative 3′ acceptor sites (A3s), alternative transcription termination sites (ATTSs), and alternative 5ʹ acceptor sites (A5s) and gain of multiple exon skipping (MES) and alternative transcription start sites (ATSSs) (Figures 9F–H). The difference between the isoforms involved in an isoform switch may arise from changes in ATSSs, alternative splicing, and ATTSs. The relative importance of alternative splicing, ATSSs, and ATTSs is shown in Figure 9I. The top genes with isoform switching, including LMNB2, RHBDL1, TRAF3IP3, VGLL4, and NFIB, are shown in Figures 9J–N.

## Discussion

ACC has been traditionally subdivided into 3 histological groups (cribriform, tubular, and solid) based on the cellular components of the tumor (Perez et al., 2006). Because of its rarity and distinctive clinical features, further exploration of the underlying mechanisms in ACC is imperative. With the rapid development of next-generation high-throughput sequencing technology (Shendure, 2008), RNA-seq has become a new and important means of gene expression and transcriptome analysis, and it can quickly and comprehensively reveal the sequence information of almost all RNA transcripts in tissues. In our study, we assessed multiple transcriptomic factors of ACC, including DEGs, gene fusions, cancer germline antigens, and different isoforms of mRNA, by analyzing 15 ACC tissues and 13 paired normal tissues.

In our study, the top 10 upregulated DEGs were COL27A1, APBA2, TUBA1A, TBX1, FNDC1, PLSCR3, FABP7, TP53, ABCC1, and EN1, while the top 10 downregulated DEGs were CST2, SMR3B, KLK1, CTBS, DHRS2, STATH, SH3BGRL2, HTN1, SLC13A5, and SLC9A1. Research has shown that EN1 and FNDC1 are significantly highly methylated genes in ACC (Bell et al., 2011). In addition, EN1 has been confirmed to be involved in cell-substrate adhesion and neural guidance and is a potential biomarker for a poor prognosis in ACC (Bell et al., 2012; Lin et al., 2022). Supplementary Table S1 shows the relevant functions of these top 20 upregulated and downregulated DE mRNAs in detail. Studies have confirmed that VEGF could promote the progression of tumors by inducing the formation of tumor vessels, and the high expression of VEGF was related to a poor prognosis in ACC patients (Liu et al., 2022). The MAPK/ERK and Wnt/β-catenin signaling pathways have been proven to be related to the progression and metastasis of ACC, and blocking these pathways regulates the migration and invasion of ACC cells. MicroRNA-181a suppresses salivary adenoid cystic carcinoma metastasis by targeting the MAPK–Snai2 pathway (Ji et al., 2020). In ACC, the mutation rate of PIK3CA is 5%–22%, and this mutation or activation of the PI3K/AKT pathway by upstream signals (EGFR, HIF-1 α, etc.) could promote the invasion, progression and metastasis of ACC cells (Sun et al., 2021; Han et al., 2022). The mutation rate of p53 in ACC was 8%, and its expression was strongly correlated with solid ACC subtypes. The mutant p53 protein not only lost its original function of inhibiting cancer but also induced excessive cell proliferation, leading to cellular malignant transformation and increased tumor invasion. Compared to ACC patients without p53 mutations, those with p53 mutations had a higher probability of metastasis, local recurrence and nerve infiltration and a shorter median survival period (Adderley et al., 2021). Functional annotation analysis showed that the upregulated DEGs were significantly enriched in the GO BP term axonogenesis. The genes involved in axonogenesis are mainly those encoding neurotrophins and their receptors, including NGF, BDNF, NTF4, and NTRK3. In previous studies, NGF, BDNF, and NTRK3 were found to be overexpressed in ACC tissues and were significantly correlated with invasion, metastasis and a poor prognosis. NGF is a member of the NGF-beta family and encodes a secreted protein that stimulates nerve growth activity and is involved in the regulation of growth and the differentiation of sympathetic and certain sensory neurons. NGF was significantly overexpressed in ACC tissues and was more strongly stained in tumors with PNI than in those without PNI. In addition, high NGF expression leads to a worse prognosis (Hao et al., 2010). BDNF encodes a protein belonging to the nerve growth factor family. BDNF is highly expressed in ACC, and the BDNF/TkB axis promotes PNI and a poor prognosis by inducing EMT progression (Jia et al., 2015). NTRK3 encodes TrkC, a member of the neurotrophic tyrosine receptor kinase family, and blocking the autocoline NT-3/TrkC signaling pathway can suppress ACC nerve invasion and growth (Ivanov et al., 2013). Neurotrophin-4 (NTF4) belongs to the family of neurotrophic factors (NTFs), which are most commonly known for their roles in the nervous system (Shen et al., 2010). BDNF and NT-4/5 can stimulate resistance to apoptosis in breast cancer cells through p75NTR and TrkB-T1 and then promote tumor progression (Vanhecke et al., 2011). NTF4 promotes cell invasion and a poor prognosis in colorectal cancer through autophagy regulation (Yang et al., 2020). However, the function of NTF4 in ACC is unclear. The importance of NTF4 in ACC needs to be further explored. Furthermore, leukocyte migration has been found to be downregulated in ACCs, and the downregulation of leukocyte migration has been found to be significantly correlated with the downregulation of chemokines, including CCL5, CCL19, CCL20, CCL21, CXCL6, and CXCL12. It has been reported that the CCL5/CCR5 axis could promote the migration, invasion and PNI of ACC (Gao et al., 2018). CXCL12/CXCR4 participate in the course of EMT and Schwann-like differentiation in PNI and can promote ACC cell invasion and PNI through the Twist/S100A4 axis (Zhang et al., 2021). In addition, CCL20 enhances the anticancer response of the immune system by recruiting dendritic cells (Bonnotte et al., 2004). The increased expression of inflammatory CCL19 and CCL21 leads to the infiltration of tumor infiltrating lymphocytes (TILs) and improves the anticancer effect and prognosis of multiple types of cancer, including liver cancer, breast cancer, and colorectal cancer (Sharma et al., 2000; Liang et al., 2007; Wu et al., 2011). In summary, these downregulated chemokines are related to PNI, tumor progression and a type IV (immune tolerance) immune microenvironment in ACC.

In addition, the downregulated DEGs in ACCs were mainly enriched in BP terms related to the adaptive immune response, lymphocyte-mediated immunity, and the humoral immune response. The downregulated DEGs associated with the humoral immune response mainly included B-cell-related and immunoglobulin genes. The downregulated DEGs associated with the adaptive immune response mainly included T-cell-related genes, especially CD8^+^ T-cell-related genes. These results suggest that lymphocytes play a crucial role in ACC. In addition, in our analysis, eight major immune cell types (CD3^+^ T-cells, CD8^+^ T-cells, cytotoxic lymphocytes, NK cells, B lymphocytes, monocytic lineage cells, myeloid dendritic cells and neutrophils) mostly showed low infiltration, and M1/M2 macrophage analysis suggested downregulation of overall inflammation in ACC tissue. A microenvironment with low CD8^+^ tumor-infiltrating lymphocyte (TIL) levels was considered a type IV (immune tolerant) microenvironment, which was consistent with a previous report (Mosconi et al., 2019). The immune environment in ACC tissue is an important factor determining cell proliferation and the efficacy of immunotherapy, chemotherapy and radiotherapy (Demaria et al., 2015; Galluzzi et al., 2015; Weichselbaum et al., 2017). ACC recurrence was found to be associated with low CD8^+^ TIL counts (Mosconi et al., 2019). In our immunohistochemistry analysis, samples from patients with ACC were analyzed for the expression of CD3, CD4, CD8, and CD19, and the results suggested that the ACC microenvironment exhibits low immunogenicity, as represented by low T and B-cells. The type IV microenvironment may help the tumor escape from the immune system and partly explain the poor prognosis of ACC patients.

In ACC, the recurrent t (6; 9) (q22-23; p23-24) (Nordkvist et al., 1994) translocation in adenoid cystic carcinoma results in a novel fusion of the MYB proto-oncogene with the transcription factor gene NFIB (Persson et al., 2009). In contrast, the MYB-NFIB fusion is not expressed in non-adenoid cystic carcinoma neoplasms of the head and neck, confirming the high specificity of the MYB-NFIB fusion, which has been used as a diagnostic marker for ACC (Toper and Sarioglu, 2021). Moreover, in 2016, a translocation involving MYBL1 and NFIB on chromosomes 8 and 9 [t (8; 9)] was found in most ACC patients without MYB-NFIB fusion (Mitani et al., 2016). The activation of MYB is considered a potential therapeutic target for ACC. Tumor progression can be prevented by silencing MYB, targeting the downstream effector of this gene, and blocking the protein‒protein interaction in the transcriptional complex (Liu et al., 2018). In our study, the MYB-NFIB fusion gene appeared in 8 of 15 (53.3%) ACC tissues, and the MYBL1-NFIB fusion gene appeared in 3 of 15 (20%) ACC tissues; the rates were slightly higher than those in the literature [45% (9/20) and 15% (3/20), respectively, as determined by RNA-seq using formalin-fixed, paraffin-embedded (FFPE) tissues] (Shibata et al., 2021). It is worth mentioning that a new gene fusion, the TVP23C-CDRT4 fusion, was found in our transcriptome sequencing study. TVP23C-CDRT4 gene fusions include different anatomical regions of chromosome 17 and were found in 3 of 15 (20%) ACC tissues. TVP23C is involved in protein secretion and vesicle-mediated transport. For patients with colorectal cancer, a high level of TVP23C in plasma indicates a longer survival period (Holm et al., 2019). CDRT4 is upregulated in breast cancer cells (Yellapu et al., 2022). However, research on TVP23C-CDRT4 gene fusion is very rare. This study is the first report of the fusion gene in ACCs. In addition, the expression of TVP23C and CDRT4 is upregulated mainly in solid-type ACCs (2/3), which indicates that TVP23C and CDRT4 may be useful for distinguishing solid-type ACC from the cribriform and tubular types of ACC. The TVP23C-CDRT4 fusion gene may promote the development of malignancy, and our results provide new targets for personalized treatment, although they need to be confirmed by further research.

Cancer cells can evade immune surveillance, which allows them to grow and metastasize. One mechanism of immune escape during cancer development is the downregulation of HLA class I molecule expression (Marincola et al., 2000; Seliger et al., 2002; Raffaghello et al., 2005; Lopez-Albaitero et al., 2006). HLA class I molecules play a central role in cell-mediated immunity, especially as antigen-presenting molecules for cytotoxic T lymphocytes (CTLs), which recognize tumor antigen-bound peptides presented on the cell surface through HLA class I molecules and kill the target cancer cell (Khong and Restifo, 2002; Bubenik, 2004). HLA class I expression appears to be lost or downregulated on the tumor cell surface, which could represent a mechanism by which neoplastic cells escape killing by CTLs, allowing tumor dissemination and metastasis (Pistillo et al., 2000). HLA class I antigen downregulation has been used as a prognostic biomarker in esophageal cancer (Hosch et al., 1997), melanoma (Kageshita et al., 1999), lung cancer (Kikuchi et al., 2007), ovarian cancer (Vitale et al., 2005), renal cell carcinoma (Kitamura et al., 2007), bladder cancer (Homma et al., 2009), and oral squamous cell carcinoma (Koike et al., 2020) and has improved the survival of patients. In our study, the expression levels of class I HLA molecules, which are responsible for antigen presentation to tumor-killing T-cells, and the expression levels of class II HLA molecules, which are responsible for antigen presentation to helper T-cells, were decreased in some patients. HLA class I molecules play a central role in anticancer immunity, but their value in ACC remains unclear. Our data suggest that tumor cells may escape tumor immunity by downregulating HLA genes.

\PRAME, first described as a melanoma antigen (Ikeda et al., 1997), is known to repress retinoic acid receptor signaling and to repress the transcription of genes involved in growth arrest, differentiation, and apoptosis in melanoma models (Epping et al., 2005). Subsequently, numerous studies reported that overexpression of PRAME was significantly correlated with clinicopathological features in malignant cancers, including medulloblastoma (Orlando et al., 2018), lung adenocarcinoma (Pan et al., 2017), uveal melanoma (Gezgin et al., 2017), high-grade serous cancer (Zhang et al., 2016), myxoid liposarcoma (Iura et al., 2015), osteosarcoma (Tan et al., 2012), bladder cancer (Dyrskjot et al., 2012), breast carcinoma (Epping et al., 2008), and solitary fibrous tumors (Wang et al., 2021). In our research, PRAME was obviously upregulated in ACC tissues. However, the role of PRAME in the occurrence and development of ACC remains unknown. This is the first time that PRAME has been found to be upregulated in ACC. It is common knowledge that the efficacy of chemotherapy and targeted drugs is poor in recurrent, unresectable, or metastatic ACC (Papaspyrou et al., 2011; Hanna et al., 2020). In addition, ACC tends to exhibit low levels of TILs (Mosconi et al., 2019). These features suggest that ACC may not be an ideal candidate for a lymphocyte immune checkpoint inhibitor-based approach to immunotherapy. Therefore, PRAME may be an attractive target for immunotherapy against ACC.

Alternative splicing is a ubiquitous regulatory mechanism of gene expression that allows the generation of more than one unique mRNA species from a single gene (Baralle and Giudice, 2017). Alternative splicing provides transcriptional plasticity by controlling which RNA isoforms are expressed at a given time in a given cell type. Cancer cells subvert this process to produce isoforms that benefit cell proliferation or migration or enable escape from cell death (Urbanski et al., 2018). Compared to normal tissues, ACCs have a significantly higher fraction of switches resulting in loss of complete ORFs, domains, and IDRs; intron retention; and shortened ORFs and have a significantly higher fraction of switches resulting in loss of A3s, ATTSs, and A5s and gain of MES and ATSSs. The differences between the isoforms involved in an isoform switch can arise via changes in three distinct biological factors: ATSSs, alternative splicing, and ATTSs. The top genes with isoform switching were LMNB2, RHBDL1, TRAF3IP3, VGLL4, and NFIB. Among them, LMNB2 is significantly upregulated in ACCs, and it is probably upregulated by mRNA isoforms with coding functions. VGLL4 is a transcription factor and is upregulated in ACCs. It has three isoforms with coding functions and a non-coding isoform. One of the isoforms with coding function is specifically upregulated in ACCs, and one domain of this isoform is different from that in other subtypes, which may have an impact on its transcriptional regulation function.

In our study, neurotrophins and their receptors were found to be closely related to the characteristics of PNI. The downregulated genes were mainly related to the antitumor immune response in the tumor microenvironment. Eight major immune cell types, CD3^+^ T-cells, CD8^+^ T-cells, cytotoxic lymphocytes, NK cells, B lymphocytes, monocytic lineage cells, myeloid dendritic cells and neutrophils, mostly showed low infiltration, and M1/M2 macrophage analysis suggested downregulation of overall inflammation in ACC tissue. Furthermore, in our preliminary study, T-cells and B-cells also showed low infiltration in ACC tissues by immunohistochemistry. Moreover, we found a new fusion gene, TVP23C-CDRT4, that may be a specific marker for solid-type ACC. We also assessed the expression of cancer germline antigens. PRAME was significantly upregulated in ACC tissues and may be an attractive target for immunotherapy against ACC. We also performed isoform and DEG analyses.

## Conclusion

Through RNA-seq of 15 ACC tissues and 13 paired normal tissues, we found significantly upregulated and downregulated genes and pathways in ACC, and T-cells, B-cells and NK cells showed low infiltration in ACC tissues. In addition to the classic gene fusions MYB-NFIB and MYBL1-NFIB, a new gene fusion, TVP23C-CDRT4, was also detected in 3 ACC tissues, and the expression of TVP23C and CDRT4 was significantly upregulated in solid-type ACC. Screening of cancer germline antigens showed that PRAME was significantly upregulated, while antigen-presenting human leukocyte antigen (HLA) genes were downregulated.

## Future perspectives

We found a new fusion gene, TVP23C-CDRT4, that may be a specific marker for solid-type ACC. PRAME is significantly upregulated in ACC tissues and may be an attractive target for immunotherapy against ACC. This study provides an accurate transcriptome analysis of ACC and a basis for the study of the molecular mechanism of ACC.

## Data Availability

Readers can obtain the data through NCBI database (BioProject accession number PRJNA900701).

## Abbreviations

ACC, adenoid cystic carcinoma; ATSSs, alternative transcription start sites; ATTSs, alternative transcription termination sites; DEGs, differentially expressed genes; GO, gene ontology; GSEA, gene set enrichment analysis; HLA, human leukocyte antigen; IDR, intrinsically disordered region; KEGG, kyoto encyclopedia of genes and genomes; MES, multiple exon skipping; NSG, normal salivary gland; ORF, open reading frame; PC1, first principle component; PC2, second principle component; PNI, perineural invasion.

## References

[B1] Adderley H. Rack S. Hapuarachi B. Feeney L. Morgan D. Hussell T. (2021). The utility of TP53 and PIK3CA mutations as prognostic biomarkers in salivary adenoid cystic carcinoma. Oral Oncol. 113, 105095. 10.1016/j.oraloncology.2020.105095 33290961

[B2] Amit M. Binenbaum Y. Trejo-Leider L. Sharma K. Ramer N. Ramer I. (2015). International collaborative validation of intraneural invasion as a prognostic marker in adenoid cystic carcinoma of the head and neck. Head. Neck 37 (7), 1038–1045. 10.1002/hed.23710 24710845

[B3] Atallah S. Casiraghi O. Fakhry N. Wassef M. Uro-Coste E. Espitalier F. (2020). A prospective multicentre REFCOR study of 470 cases of head and neck adenoid cystic carcinoma: Epidemiology and prognostic factors. Eur. J. Cancer 130, 241–249. 10.1016/j.ejca.2020.01.023 32171628

[B4] Baralle F. E. Giudice J. (2017). Alternative splicing as a regulator of development and tissue identity. Nat. Rev. Mol. Cell. Biol. 18 (7), 437–451. 10.1038/nrm.2017.27 28488700 PMC6839889

[B5] Bell A. Bell D. Weber R. S. El-Naggar A. K. (2011). CpG island methylation profiling in human salivary gland adenoid cystic carcinoma. Cancer 117 (13), 2898–2909. 10.1002/cncr.25818 21692051 PMC3123690

[B6] Bell D. Bell A. Roberts D. Weber R. S. El-Naggar A. K. (2012). Developmental transcription factor EN1--a novel biomarker in human salivary gland adenoid cystic carcinoma. Cancer 118 (5), 1288–1292. 10.1002/cncr.26412 21800291 PMC3208084

[B7] Bonnotte B. Crittenden M. Larmonier N. Gough M. Vile R. G. (2004). MIP-3alpha transfection into a rodent tumor cell line increases intratumoral dendritic cell infiltration but enhances (facilitates) tumor growth and decreases immunogenicity. J. Immunol. 173 (8), 4929–4935. 10.4049/jimmunol.173.8.4929 15470034

[B8] Bubenik J. (2004). MHC class I down-regulation: Tumour escape from immune surveillance? (Review). Int. J. Oncol. 25 (2), 487–491. 10.3892/ijo.25.2.487 15254748

[B9] de Morais E. F. da Silva L. P. Moreira D. G. L. Mafra R. P. Rolim L. S. A. de Moura Santos E. (2021). Prognostic factors and survival in adenoid cystic carcinoma of the head and neck: A retrospective clinical and histopathological analysis of patients seen at a cancer center. Head. Neck Pathol. 15 (2), 416–424. 10.1007/s12105-020-01210-7 32779101 PMC8134621

[B10] Demaria S. Golden E. B. Formenti S. C. (2015). Role of local radiation therapy in cancer immunotherapy. Jama Oncol. 1 (9), 1325–1332. 10.1001/jamaoncol.2015.2756 26270858

[B11] Dyrskjot L. Zieger K. Lildal T. K. Reinert T. Gruselle O. Coche T. (2012). Expression of MAGE-A3, NY-ESO-1, LAGE-1 and PRAME in urothelial carcinoma. Br. J. Cancer 107 (1), 116–122. 10.1038/bjc.2012.215 22596240 PMC3389414

[B12] Epping M. T. Wang L. M. Edel M. J. Carlee L. Hernandez M. Bernards R. (2005). The human tumor antigen PRAME is a dominant repressor of retinoic acid receptor signaling. Cell. 122 (6), 835–847. 10.1016/j.cell.2005.07.003 16179254

[B13] Epping M. T. Hart A. A. M. Glas A. M. Krijgsman O. Bernards R. (2008). PRAME expression and clinical outcome of breast cancer. Br. J. Cancer 99 (3), 398–403. 10.1038/sj.bjc.6604494 18648365 PMC2527791

[B14] Fang Y. Peng Z. Wang Y. Gao K. Liu Y. Fan R. (2022). Current opinions on diagnosis and treatment of adenoid cystic carcinoma. Oral Oncol. 130, 105945. 10.1016/j.oraloncology.2022.105945 35662026

[B15] Ferrarotto R. Mitani Y. McGrail D. J. Li K. Karpinets T. V. Bell D. (2021). Proteogenomic analysis of salivary adenoid cystic carcinomas defines molecular subtypes and identifies therapeutic targets. Clin. Cancer Res. 27 (3), 852–864. 10.1158/1078-0432.CCR-20-1192 33172898 PMC7854509

[B16] Galluzzi L. Buque A. Kepp O. Zitvogel L. Kroemer G. (2015). Immunological effects of conventional chemotherapy and targeted anticancer agents. Cancer Cell. 28 (6), 690–714. 10.1016/j.ccell.2015.10.012 26678337

[B17] Gao M. Hao Y. Huang M. X. Ma D. Q. Luo H. Y. Gao Y. (2013). Clinicopathological study of distant metastases of salivary adenoid cystic carcinoma. Int. J. Oral Maxillofac. Surg. 42 (8), 923–928. 10.1016/j.ijom.2013.04.006 23706387

[B18] Gao T. Shen Z. Y. Ma C. Li Y. Kang X. F. Sun M. Y. (2018). The CCL5/CCR5 chemotactic pathway promotes perineural invasion in salivary adenoid cystic carcinoma. J. Oral Maxillofac. Surg. 76 (8), 1708–1718. 10.1016/j.joms.2018.02.009 29549020

[B19] Gezgin G. Luk S. J. Cao J. F. Dogrusoz M. van der Steen D. M. Hagedoorn R. S. (2017). PRAME as a potential target for immunotherapy in metastatic uveal melanoma. Jama Ophthalmol. 135 (6), 541–549. 10.1001/jamaophthalmol.2017.0729 28448663 PMC5509351

[B20] Haas B. J. Dobin A. Li B. Stransky N. Pochet N. Regev A. (2019). Accuracy assessment of fusion transcript detection via read-mapping and de novo fusion transcript assembly-based methods. Genome Biol. 20 (1), 213. 10.1186/s13059-019-1842-9 31639029 PMC6802306

[B21] Han N. Li X. Wang Y. Li H. Zhang C. Zhao X. (2022). HIF-1α induced NID1 expression promotes pulmonary metastases via the PI3K-AKT pathway in salivary gland adenoid cystic carcinoma. Oral Oncol. 131, 105940. 10.1016/j.oraloncology.2022.105940 35689951

[B22] Hanna G. J. Bae J. E. Lorch J. H. Schoenfeld J. D. Margalit D. N. Tishler R. B. (2020). Long-term outcomes and clinicogenomic correlates in recurrent, metastatic adenoid cystic carcinoma. Oral Oncol. 106, 104690. 10.1016/j.oraloncology.2020.104690 32283496

[B23] Hao L. Xiao-lin N. Qi C. Yi-ping Y. Jia-quan L. Yan-ning L. (2010). Nerve growth factor and vascular endothelial growth factor: Retrospective analysis of 63 patients with salivary adenoid cystic carcinoma. Int. J. Oral Sci. 2 (1), 35–44. 10.4248/IJOS10005 20690417 PMC3475596

[B24] Holm M. Joenväärä S. Saraswat M. Mustonen H. Tohmola T. Ristimäki A. (2019). Identification of several plasma proteins whose levels in colorectal cancer patients differ depending on outcome. FASEB Bioadv 1 (12), 723–730. 10.1096/fba.2019-00062 32123817 PMC6996405

[B25] Homma I. Kitamura H. Torigoe T. Tanaka T. Sato E. Hirohashi Y. (2009). Human leukocyte antigen class I down-regulation in muscle-invasive bladder cancer: Its association with clinical characteristics and survival after cystectomy. Cancer Sci. 100 (12), 2331–2334. 10.1111/j.1349-7006.2009.01329.x 19751235 PMC11158967

[B26] Hong M. Y. Tao S. Zhang L. Diao L. T. Huang X. M. Huang S. H. (2020). RNA sequencing: New technologies and applications in cancer research. J. Hematol. Oncol. 13 (1), 166. 10.1186/s13045-020-01005-x 33276803 PMC7716291

[B27] Hosch S. B. Izbicki J. R. Pichlmeier U. Stoecklein N. Niendorf A. Knoefel W. T. (1997). Expression and prognostic significance of immunoregulatory molecules in esophageal cancer. Int. J. Cancer 74 (6), 582–587. 10.1002/(sici)1097-0215(19971219)74:6<582:aid-ijc4>3.0.co;2-q 9421352

[B28] Ikeda H. Lethe B. Lehmann F. VanBaren N. Baurain J. F. DeSmet C. (1997). Characterization of an antigen that is recognized on a melanoma showing partial HLA loss by CTL expressing an NK inhibitory receptor. Immunity 6 (2), 199–208. 10.1016/s1074-7613(00)80426-4 9047241

[B29] Iura K. Kohashi K. Hotokebuchi Y. Ishii T. Maekawa A. Yamada Y. (2015). Cancer-testis antigens PRAME and NY-ESO-1 correlate with tumour grade and poor prognosis in myxoid liposarcoma. J. Pathology Clin. Res. 1 (3), 144–159. 10.1002/cjp2.16 PMC4939879 27499900

[B30] Ivanov S. V. Panaccione A. Brown B. Guo Y. Moskaluk C. A. Wick M. J. (2013). TrkC signaling is activated in adenoid cystic carcinoma and requires NT-3 to stimulate invasive behavior. Oncogene 32 (32), 3698–3710. 10.1038/onc.2012.377 23027130

[B31] Jang S. Patel P. N. Kimple R. J. McCulloch T. M. (2017). Clinical outcomes and prognostic factors of adenoid cystic carcinoma of the head and neck. Anticancer Res. 37 (6), 3045–3052. 10.21873/anticanres.11659 28551643 PMC7238770

[B32] Ji H. Ding X. Zhang W. Zheng Y. Du H. Zheng Y. (2020). Claudin-7 inhibits proliferation and metastasis in salivary adenoid cystic carcinoma through wnt/β-catenin signaling. Cell. Transpl. 29, 963689720943583. 10.1177/0963689720943583 PMC7563826 32749148

[B33] Jia S. Wang W. Hu Z. Shan C. Wang L. Wu B. (2015). BDNF mediated TrkB activation contributes to the EMT progression and the poor prognosis in human salivary adenoid cystic carcinoma. Oral Oncol. 51 (1), 64–70. 10.1016/j.oraloncology.2014.10.008 25456007

[B34] Kageshita T. Hirai S. Ono T. Hicklin D. J. Ferrone S. (1999). Down-regulation of HLA class I antigen-processing molecules in malignant melanoma - association with disease progression. Am. J. Pathology 154 (3), 745–754. 10.1016/s0002-9440(10)65321-7 PMC1866429 10079252

[B35] Khong H. T. Restifo N. P. (2002). Natural selection of tumor variants in the generation of "tumor escape" phenotypes. Nat. Immunol. 3 (11), 999–1005. 10.1038/ni1102-999 12407407 PMC1508168

[B36] Kikuchi E. Yamazaki K. Torigoe T. Cho Y. Miyamoto M. Oizumi S. (2007). HLA class I antigen expression is associated with a favorable prognosis in early stage non-small cell lung cancer. Cancer Sci. 98 (9), 1424–1430. 10.1111/j.1349-7006.2007.00558.x 17645781 PMC11159758

[B37] Kitamura H. Honma I. Torigoe T. Asanuma H. Sato N. Tsukamoto T. (2007). Down-regulation of HLA class I antigen is an independent prognostic factor for clear cell renal cell carcinoma. J. Urology 177 (4), 1269–1272. 10.1016/j.juro.2006.11.082 17382705

[B38] Koike K. Dehari H. Shimizu S. Nishiyama K. Sonoda T. Ogi K. (2020). Prognostic value of HLA class I expression in patients with oral squamous cell carcinoma. Cancer Sci. 111 (5), 1491–1499. 10.1111/cas.14388 32167621 PMC7226222

[B39] Liang C. M. Zhong C. P. Sun R. X. Liu B. B. Huang C. Qin J. (2007). Local expression of secondary lymphoid tissue chemokine delivered by adeno-associated virus within the tumor bed stimulates strong anti-liver tumor immunity. J. Virol. 81 (17), 9502–9511. 10.1128/JVI.00208-07 17567706 PMC1951415

[B40] Lin Q. Fang Z. Sun J. Chen F. Ren Y. Fu Z. (2022). Single-cell transcriptomic analysis of the tumor ecosystem of adenoid cystic carcinoma. Front. Oncol. 12, 1063477. 10.3389/fonc.2022.1063477 36465348 PMC9714264

[B41] Liu X. Xu Y. Han L. Yi Y. (2018). Reassessing the potential of myb-targeted anti-cancer therapy. J. Cancer 9 (7), 1259–1266. 10.7150/jca.23992 29675107 PMC5907674

[B42] Liu H. Chen L. Wang C. Zhou H. (2022). The expression and significance of vascular endothelial growth factor A in adenoid cystic carcinoma of palatal salivary gland. Eur. Arch. Otorhinolaryngol. 279 (12), 5869–5875. 10.1007/s00405-022-07502-8 35781742

[B43] Lopez-Albaitero A. Nayak J. V. Ogino T. Machandia A. Gooding W. Deleo A. B. (2006). Role of antigen-processing machinery in the *in vitro* resistance of squamous cell carcinoma of the head and neck cells to recognition by CTL. J. Immunol. 176 (6), 3402–3409. 10.4049/jimmunol.176.6.3402 16517708

[B44] Love M. I. Huber W. Anders S. (2014). Moderated estimation of fold change and dispersion for RNA-seq data with DESeq2. Genome Biol. 15 (12), 550. 10.1186/s13059-014-0550-8 25516281 PMC4302049

[B45] Margulies M. Egholm M. Altman W. E. Attiya S. Bader J. S. Bemben L. A. (2005). Genome sequencing in microfabricated high-density picolitre reactors. Nature 437 (7057), 376–380. 10.1038/nature03959 16056220 PMC1464427

[B46] Marincola F. M. Jaffee E. M. Hicklin D. J. Ferrone S. (2000). Escape of human solid tumors from T-cell recognition: Molecular mechanisms and functional significance. Adv. Immunol. 74, 181–273. 10.1016/s0065-2776(08)60911-6 10605607

[B47] Meyerson M. Gabriel S. Getz G. (2010). Advances in understanding cancer genomes through second-generation sequencing. Nat. Rev. Genet. 11 (10), 685–696. 10.1038/nrg2841 20847746

[B48] Mitani Y. Liu B. Rao P. H. Borra V. J. Zafereo M. Weber R. S. (2016). Novel MYBL1 gene rearrangements with recurrent MYBL1-NFIB fusions in salivary adenoid cystic carcinomas lacking t(6;9) translocations. Clin. Cancer Res. 22 (3), 725–733. 10.1158/1078-0432.Ccr-15-2867-t 26631609 PMC4807116

[B49] Morozova O. Marra M. A. (2008). Applications of next-generation sequencing technologies in functional genomics. Genomics 92 (5), 255–264. 10.1016/j.ygeno.2008.07.001 18703132

[B50] Mosconi C. de Arruda J. A. A. de Farias A. C. R. Oliveira G. A. Q. de Paula H. M. Fonseca F. P. (2019). Immune microenvironment and evasion mechanisms in adenoid cystic carcinomas of salivary glands. Oral Oncol. 88, 95–101. 10.1016/j.oraloncology.2018.11.028 30616805

[B51] Nordkvist A. Mark J. Gustafsson H. Bang G. Stenman G. (1994). Nonrandom chromosome rearrangements in adenoid cystic carcinoma of the salivary-glands. Genes. Chromosomes Cancer 10 (2), 115–121. 10.1002/gcc.2870100206 7520264

[B52] Orlando D. Miele E. De Angelis B. Guercio M. Boffa I. Sinibaldi M. (2018). Adoptive immunotherapy using PRAME-specific T cells in medulloblastoma. Cancer Res. 78 (12), 3337–3349. 10.1158/0008-5472.Can-17-3140 29615432

[B53] Pan S. H. Su K. Y. Spiessens B. Kusuma N. Delahaye N. F. Gruselle O. (2017). Gene expression of MAGE-A3 and PRAME tumor antigens and EGFR mutational status in Taiwanese non-small cell lung cancer patients. Asia-Pacific J. Clin. Oncol. 13 (5), E212–E223. 10.1111/ajco.12586 27519286

[B54] Papaspyrou G. Hoch S. Rinaldo A. Rodrigo J. P. Takes R. P. van Herpen C. (2011). Chemotherapy and targeted therapy in adenoid cystic carcinoma of the head and neck: A review. Head Neck-Journal Sci. Specialties Head Neck 33 (6), 905–911. 10.1002/hed.21458 20652885

[B55] Perez D. E. D. Alves F. D. Nishimoto I. N. de Almeida O. P. Kowalski L. P. (2006). Prognostic factors in head and neck adenoid cystic carcinoma. Oral Oncol. 42 (2), 139–146. 10.1016/j.oraloncology.2005.06.024 16249115

[B56] Persson M. Andren Y. Mark J. Horlings H. M. Persson F. Stenman G. (2009). Recurrent fusion of MYB and NFIB transcription factor genes in carcinomas of the breast and head and neck. Proc. Natl. Acad. Sci. U. S. A. 106 (44), 18740–18744. 10.1073/pnas.0909114106 19841262 PMC2773970

[B57] Pistillo M. P. Nicolo G. Salvi S. Capanni P. Perdelli L. Pasciucco G. (2000). Biochemical analysis of HLA class I subunits expression in breast cancer tissues. Hum. Immunol. 61 (4), 397–407. 10.1016/s0198-8859(99)00179-2 10715517

[B58] Raffaghello L. Prigione I. Airoldi I. Camoriano M. Morandi F. Bocca P. (2005). Mechanisms of immune evasion of human neuroblastoma. Cancer Lett. 228 (1-2), 155–161. 10.1016/j.canlet.2004.11.064 15923080

[B59] Ren S. C. Peng Z. Y. Mao J. H. Yu Y. W. Yin C. J. Gao X. (2012). RNA-seq analysis of prostate cancer in the Chinese population identifies recurrent gene fusions, cancer-associated long noncoding RNAs and aberrant alternative splicings. Cell. Res. 22 (5), 806–821. 10.1038/cr.2012.30 22349460 PMC3343650

[B60] Ross J. S. Gay L. M. Wang K. Vergilio J. A. Suh J. Ramkissoon S. (2017). Comprehensive genomic profiles of metastatic and relapsed salivary gland carcinomas are associated with tumor type and reveal new routes to targeted therapies. Ann. Oncol. 28 (10), 2539–2546. 10.1093/annonc/mdx399 28961851 PMC5834110

[B61] Seliger B. Cabrera T. Garrido F. Ferrone S. (2002). HLA class I antigen abnormalities and immune escape by malignant cells. Seminars Cancer Biol. 12 (1), 3–13. 10.1006/scbi.2001.0404 11926409

[B62] Sharma S. Stolina M. Luo J. Strieter R. M. Burdick M. Zhu L. X. (2000). Secondary lymphoid tissue chemokine mediates T cell-dependent antitumor responses *in vivo* . J. Immunol. 164 (9), 4558–4563. 10.4049/jimmunol.164.9.4558 10779757

[B63] Shen Y. F. Inoue N. Heese K. (2010). Neurotrophin-4 (Ntf4) mediates neurogenesis in mouse embryonic neural stem cells through the inhibition of the signal transducer and activator of transcription-3 (Stat3) and the modulation of the activity of protein kinase B. Cell. Mol. Neurobiol. 30 (6), 909–916. 10.1007/s10571-010-9520-1 20407817 PMC11498831

[B64] Shendure J. (2008). The beginning of the end for microarrays? Nat. Methods 5 (7), 585–587. 10.1038/nmeth0708-585 18587314

[B65] Shibata E. Morita K.-I. Kayamori K. Tange S. Shibata H. Harazono Y. (2021). Detection of novel fusion genes by next-generation sequencing-based targeted RNA sequencing analysis in adenoid cystic carcinoma of head and neck. Oral Surg. oral Med. oral pathology oral radiology 132 (4), 426–433. 10.1016/j.oooo.2021.03.020 34413003

[B66] Sood S. McGurk M. Vaz F. (2016). Management of salivary gland tumours: United Kingdom national multidisciplinary guidelines. J. Laryngology Otology 130, S142–S149. 10.1017/s0022215116000566 PMC4873929 27841127

[B67] Sun B. Wang Y. Sun J. Zhang C. Xia R. Xu S. (2021). Establishment of patient-derived xenograft models of adenoid cystic carcinoma to assess pre-clinical efficacy of combination therapy of a PI3K inhibitor and retinoic acid. Am. J. Cancer Res. 11 (3), 773–792. 33791153 PMC7994170

[B68] Sung H. Ferlay J. Siegel R. L. Laversanne M. Soerjomataram I. Jemal A. (2021). Global cancer statistics 2020: GLOBOCAN estimates of incidence and mortality worldwide for 36 cancers in 185 countries. CA Cancer J. Clin. 71 (3), 209–249. 10.3322/caac.21660 33538338

[B69] Szanto P. A. Luna M. A. Tortoledo M. E. White R. A. (1984). Histologic grading of adenoid cystic carcinoma of the salivary-glands. Cancer 54 (6), 1062–1069. 10.1002/1097-0142(19840915)54:6<1062:Aid-cncr2820540622>3.0.Co;2-e 6088017

[B70] Tan P. X. Zou C. Y. Yong B. C. Han J. Zhang L. J. Su Q. (2012). Expression and prognostic relevance of PRAME in primary osteosarcoma. Biochem. Biophysical Res. Commun. 419 (4), 801–808. 10.1016/j.bbrc.2012.02.110 22390931

[B71] Tang Y. L. Liang X. H. Zheng M. Zhu Z. Y. Zhu G. Q. Yang J. (2010). Expression of c-kit and Slug correlates with invasion and metastasis of salivary adenoid cystic carcinoma. Oral Oncol. 46 (4), 311–316. 10.1016/j.oraloncology.2010.02.001 20219417

[B72] Toper M. H. Sarioglu S. (2021). Molecular Pathology of salivary gland neoplasms: Diagnostic, prognostic, and predictive perspective. Adv. Anat. Pathol. 28 (2), 81–93. 10.1097/PAP.0000000000000291 33405400

[B73] Uhrig S. Ellermann J. Walther T. Burkhardt P. Froehlich M. Hutter B. (2021). Accurate and efficient detection of gene fusions from RNA sequencing data. Genome Res. 31 (3), 448–460. 10.1101/gr.257246.119 33441414 PMC7919457

[B74] Urbanski L. M. Leclair N. Anczukow O. (2018). Alternative-splicing defects in cancer: Splicing regulators and their downstream targets, guiding the way to novel cancer therapeutics. Wiley Interdiscip. Reviews-Rna 9 (4), e1476. 10.1002/wrna.1476 PMC6002934 29693319

[B75] Vanhecke E. Adriaenssens E. Verbeke S. Meignan S. Germain E. Berteaux N. (2011). Brain-Derived neurotrophic factor and neurotrophin-4/5 are expressed in breast cancer and can Be targeted to inhibit tumor cell survival. Clin. Cancer Res. 17 (7), 1741–1752. 10.1158/1078-0432.Ccr-10-1890 21350004

[B76] Vitale M. Pelusi G. Taroni B. Gobbi G. Micheloni C. Rezzani R. (2005). HLA class I antigen down-regulation in primary ovary carcinoma lesions: Association with disease stage. Clin. Cancer Res. 11 (1), 67–72. 10.1158/1078-0432.67.11.1 15671529

[B77] Vitting-Seerup K. Sandelin A. (2019). IsoformSwitchAnalyzeR: Analysis of changes in genome-wide patterns of alternative splicing and its functional consequences. Bioinformatics 35 (21), 4469–4471. 10.1093/bioinformatics/btz247 30989184

[B78] Wang Z. Gerstein M. Snyder M. (2009). RNA-seq: A revolutionary tool for transcriptomics. Nat. Rev. Genet. 10 (1), 57–63. 10.1038/nrg2484 19015660 PMC2949280

[B79] Wang W. L. Gokgoz N. Samman B. Andrulis I. L. Wunder J. S. Demicco E. G. (2021). RNA expression profiling reveals PRAME, a potential immunotherapy target, is frequently expressed in solitary fibrous tumors. Mod. Pathol. 34 (5), 951–960. 10.1038/s41379-020-00687-5 33009490

[B80] Wei H. Y. Yang M. Ke Y. G. Liu J. N. Chen Z. B. Zhao J. R. (2022). Comparative physiological and transcriptomic profiles reveal regulatory mechanisms of soft rot disease resistance in Amorphophallus spp. Physiological Mol. Plant Pathology 118, 101807. 10.1016/j.pmpp.2022.101807

[B81] Weichselbaum R. R. Liang H. Deng L. F. Fu Y. X. (2017). Radiotherapy and immunotherapy: A beneficial liaison? Nat. Rev. Clin. Oncol. 14 (6), 365–379. 10.1038/nrclinonc.2016.211 28094262

[B82] Wu S. Lu X. Zhang Z. L. Lei P. Hu P. Wang M. (2011). CC chemokine ligand 21 enhances the immunogenicity of the breast cancer cell line MCF-7 upon assistance of TLR2. Carcinogenesis 32 (3), 296–304. 10.1093/carcin/bgq265 21149644

[B83] Yang Z. Chen Y. S. Wei X. Y. Wu D. J. Min Z. J. Quan Y. J. (2020). Upregulated NTF4 in colorectal cancer promotes tumor development via regulating autophagy. Int. J. Oncol. 56 (6), 1442–1454. 10.3892/ijo.2020.5027 32236587 PMC7170041

[B84] Yellapu N. K. Ly T. Sardiu M. E. Pei D. Welch D. R. Thompson J. A. (2022). Synergistic anti-proliferative activity of JQ1 and GSK2801 in triple-negative breast cancer. BMC Cancer 22 (1), 627. 10.1186/s12885-022-09690-2 35672711 PMC9173973

[B85] Yu G. C. Wang L. G. Han Y. Y. He Q. Y. (2012). clusterProfiler: an R Package for comparing biological themes among gene clusters. Omics-a J. Integr. Biol. 16 (5), 284–287. 10.1089/omi.2011.0118 PMC3339379 22455463

[B86] Zhang W. Barger C. J. Eng K. H. Klinkebiel D. Link P. A. Omilian A. (2016). PRAME expression and promoter hypomethylation in epithelial ovarian cancer. Oncotarget 7 (29), 45352–45369. 10.18632/oncotarget.9977 27322684 PMC5216727

[B87] Zhang M. Zheng M. Dai L. Zhang W. L. Fan H. Y. Yu X. H. (2021). CXCL12/CXCR4 facilitates perineural invasion via induction of the Twist/S100A4 axis in salivary adenoid cystic carcinoma. J. Cell. Mol. Med. 25, 7901–7912. 10.1111/jcmm.16713 34170080 PMC8358865

[B88] Zhu F. Y. Chen M. X. Ye N. H. Qiao W. M. Gao B. Law W. K. (2018). Comparative performance of the BGISEQ-500 and Illumina HiSeq4000 sequencing platforms for transcriptome analysis in plants. Plant Methods 14, 69. 10.1186/s13007-018-0337-0 30123314 PMC6088413

