# Supplementary material for: Transcriptomic insights into adenoid cystic carcinoma via RNA sequencing
Source: Front Genet. 2023 Apr 21;14:1144945. doi: 10.3389/fgene.2023.1144945 (PMC10160386; doi:10.3389/fgene.2023.1144945)
Supplement: Supplementary file 1 [file Table1.DOCX]

Table.1: The clinicopathological characteristics of the 15 patients

| Variable | No. of cases |
| --- | --- |
| Age  Median  Range | 56  42-73 |
| Sex |  |
| Female | 9 |
| Male | 6 |
| Location |  |
| Maxillary sinus | 4 |
| Base of tongue | 1 |
| Palate | 8 |
| Sublingual gland | 2 |
| Pathological type |  |
| Cribriform-tubular | 9 |
| Solid | 2 |
| Cribriform | 3 |
| High-Grade transformation | 1 |
| Perineural invasion |  |
| Positive | 5 |
| Negative | 10 |
| T stage |  |
| T3 | 5 |
| T4 | 10 |
| N stage |  |
| N0 | 14 |
| N2 | 1 |
| M stage |  |
| M0 | 13 |
| M1 | 2 |
