# Supplementary material for: Transcriptomic insights into adenoid cystic carcinoma via RNA sequencing
Source: Front Genet. 2023 Apr 21;14:1144945. doi: 10.3389/fgene.2023.1144945 (PMC10160386; doi:10.3389/fgene.2023.1144945)
Supplement: Supplementary file 2 [file Table2.DOCX]

Table 2: Top 20 up and downregulated DE-mRNAs

| gene_name | log2FoldChange | padj | sig |
| --- | --- | --- | --- |
| COL27A1 | 4.905059189 | 1.25E-147 | up |
| APBA2 | 5.695475595 | 8.12E-103 | up |
| TUBA1A | 3.071934334 | 5.56E-98 | up |
| TBX1 | 5.253479631 | 1.80E-97 | up |
| FNDC1 | 8.205559027 | 2.53E-90 | up |
| PLSCR3 | 2.312919959 | 6.29E-86 | up |
| FABP7 | 6.549525418 | 2.88E-82 | up |
| TP53 | 2.431556017 | 3.67E-82 | up |
| ABCC1 | 2.789483537 | 8.20E-82 | up |
| EN1 | 11.81068084 | 2.06E-81 | up |
| MFAP2 | 4.852118182 | 4.55E-80 | up |
| MEX3A | 5.502988401 | 1.08E-79 | up |
| PRAME | 10.57626928 | 1.76E-76 | up |
| STMN1 | 4.323275057 | 4.82E-76 | up |
| TRO | 3.533004668 | 1.69E-75 | up |
| OBP2B | 8.656299902 | 3.33E-72 | up |
| ITGA9 | 4.683738203 | 4.26E-69 | up |
| TTYH1 | 6.612421301 | 6.53E-66 | up |
| SOX4 | 3.716569748 | 4.25E-65 | up |
| CASC15 | 3.099970158 | 1.51E-63 | up |
| CST2 | -13.02941499 | 3.07E-202 | down |
| SMR3B | -15.78505533 | 1.02E-168 | down |
| KLK1 | -10.66554013 | 7.85E-164 | down |
| CTBS | -2.505495637 | 1.19E-136 | down |
| DHRS2 | -11.62188281 | 2.85E-132 | down |
| STATH | -15.7089987 | 6.94E-120 | down |
| SH3BGRL2 | -5.323275759 | 1.04E-119 | down |
| HTN1 | -16.16367832 | 3.66E-116 | down |
| SLC13A5 | -9.511121749 | 1.22E-115 | down |
| SLC9A1 | -3.119844922 | 6.99E-115 | down |
| HTN3 | -16.96742223 | 6.99E-115 | down |
| PON3 | -6.48883633 | 7.48E-115 | down |
| FXYD2 | -10.55561278 | 7.48E-115 | down |
| WWC1 | -3.561034857 | 5.02E-112 | down |
| PDCD4 | -2.542553396 | 5.15E-109 | down |
| CTPS1 | -3.47144039 | 3.20E-108 | down |
| FUT8 | -3.351754045 | 2.60E-104 | down |
| ATP2B2 | -8.112838869 | 2.43E-102 | down |
| BLM | -4.766716988 | 6.01885E-100 | down |
| PRR27 | -15.16202799 | 1.38806E-97 | down |
