# Supplementary material for: Transcriptomic insights into adenoid cystic carcinoma via RNA sequencing
Source: Front Genet. 2023 Apr 21;14:1144945. doi: 10.3389/fgene.2023.1144945 (PMC10160386; doi:10.3389/fgene.2023.1144945)
Supplement: Supplementary file 3 [file DataSheet1.PDF]

The statistical data for Fig 6A

Tukey's multiple comparisons test-**CD3**	Mean Diff.	95.00% CI of diff.	Below threshold?	Summary	Adjusted P Value	
Control vs. Peritumor	4.667	-67.74 to 77.08	No	ns	0.9863	A-B
Control vs. Intratumor	163.4	91.04 to 235.9	Yes	****	<0.0001	A-C
Peritumor vs. Intratumor	158.8	86.37 to 231.2	Yes	****	<0.0001	B-C

Test details	Mean 1	Mean 2	Mean Diff.	SE of diff.	n1	n2	q	DF
Control vs. Peritumor	187.2	182.6	4.667	29.51	12	12	0.2236	33
Control vs. Intratumor	187.2	23.79	163.4	29.51	12	12	7.833	33
Peritumor vs. Intratumor	182.6	23.79	158.8	29.51	12	12	7.610	33

Tukey's multiple comparisons test-**CD4**	Mean Diff.	95.00% CI of diff.	Below threshold?	Summary	Adjusted P Value	
Control vs. Peritumor	22.48	-65.66 to 110.6	No	ns	0.8071	A-B
Control vs. Intratumor	109.7	21.55 to 197.8	Yes	*	0.0120	A-C
Peritumor vs. Intratumor	87.21	-0.9317 to 175.3	No	ns	0.0529	B-C

Test details	Mean 1	Mean 2	Mean Diff.	SE of diff.	n1	n2	q	DF
Control vs. Peritumor	126.9	104.5	22.48	35.92	12	12	0.8851	33
Control vs. Intratumor	126.9	17.25	109.7	35.92	12	12	4.319	33
Peritumor vs. Intratumor	104.5	17.25	87.21	35.92	12	12	3.434	33

Tukey's multiple comparisons test-**CD8**	Mean Diff.	95.00% CI of diff.	Below threshold?	Summary	Adjusted P Value			
Control vs. Peritumor	50.30	5.740 to 94.86	Yes	*	0.0242	A-B		
Control vs. Intratumor	142.6	98.01 to 187.1	Yes	****	<0.0001	A-C		
Peritumor vs. Intratumor	92.27	47.71 to 136.8	Yes	****	<0.0001	B-C		
Test details	Mean 1	Mean 2	Mean Diff.	SE of diff.	n1	n2	q	DF
Control vs. Peritumor	168.5	118.2	50.30	18.16	12	12	3.917	33
Control vs. Intratumor	168.5	25.90	142.6	18.16	12	12	11.10	33
Peritumor vs. Intratumor	118.2	25.90	92.27	18.16	12	12	7.185	33

Tukey's multiple comparisons test-**CD19**	Mean Diff.	95.00% CI of diff.	Below threshold?	Summary	Adjusted P Value			
Control vs. Peritumor	148.1	81.85 to 214.4	Yes	****	<0.0001	A-B		
Control vs. Intratumor	207.0	140.7 to 273.3	Yes	****	<0.0001	A-C		
Peritumor vs. Intratumor	58.87	-7.410 to 125.2	No	ns	0.0897	B-C		
Test details	Mean 1	Mean 2	Mean Diff.	SE of diff.	n1	n2	q	DF
Control vs. Peritumor	209.8	61.63	148.1	27.01	12	12	7.755	33
Control vs. Intratumor	209.8	2.760	207.0	27.01	12	12	10.84	33
Peritumor vs. Intratumor	61.63	2.760	58.87	27.01	12	12	3.082	33

The statistical data for Fig 6B

Tukey's multiple comparisons test-**CD3**	Mean Diff.	95.00% CI of diff.	Below threshold?	Summary	**Adjusted P Value**	
Normal vs. Cribriform	129.4	47.61 to 211.3	Yes	**	**0.0014**	A-B
Normal vs. Cribriform&Tubular	89.29	25.91 to 152.7	Yes	**	**0.0041**	A-C
Normal vs. Solid	28.22	-53.60 to 110.0	No	ns	**0.7703**	A-D
Cribriform vs. Cribriform&Tubular	-40.15	-129.8 to 49.48	No	ns	**0.6015**	B-C
Cribriform vs. Solid	-101.2	-204.7 to 2.284	No	ns	**0.0567**	B-D
Cribriform&Tubular vs. Solid	-61.07	-150.7 to 28.56	No	ns	**0.2568**	C-D

Tukey's multiple comparisons **test-CD4**	Mean Diff.	95.00% CI of diff.	Below threshold?	Summary	**Adjusted P Value**	
Normal vs. Cribriform	103.3	-70.33 to 276.8	No	ns	**0.3673**	A-B
Normal vs. Cribriform&Tubular	56.57	-77.89 to 191.0	No	ns	**0.6474**	A-C
Normal vs. Solid	47.95	-125.6 to 221.5	No	ns	**0.8656**	A-D
Cribriform vs. Cribriform&Tubular	-46.69	-236.8 to 143.5	No	ns	**0.9008**	B-C
Cribriform vs. Solid	-55.31	-274.9 to 164.3	No	ns	**0.8939**	B-D
Cribriform&Tubular vs. Solid	-8.620	-198.8 to 181.5	No	ns	**0.9992**	C-D

Tukey's multiple comparisons test-**CD8**	Mean Diff.	95.00% CI of diff.	Below threshold?	Summary	**Adjusted P Value**	
Normal vs. Cribriform	127.2	52.39 to 202.0	Yes	***	**0.0006**	A-B
Normal vs. Cribriform&Tubular	83.57	25.62 to 141.5	Yes	**	**0.0033**	A-C
Normal vs. Solid	91.40	16.59 to 166.2	Yes	*	**0.0133**	A-D
Cribriform vs. Cribriform&Tubular	-43.63	-125.6 to 38.31	No	ns	**0.4615**	B-C
Cribriform vs. Solid	-35.80	-130.4 to 58.83	No	ns	**0.7176**	B-D
Cribriform&Tubular vs. Solid	7.833	-74.11 to 89.78	No	ns	**0.9931**	C-D

Tukey's multiple comparisons test-**CD19**	Mean Diff.	95.00% CI of diff.	Below threshold?	Summary	**Adjusted P Value**	
Normal vs. Cribriform	179.5	65.54 to 293.5	Yes	**	**0.0014**	A-B
Normal vs. Cribriform&Tubular	164.3	76.04 to 252.6	Yes	***	**0.0002**	A-C
Normal vs. Solid	202.2	88.19 to 316.1	Yes	***	**0.0004**	A-D
Cribriform vs. Cribriform&Tubular	-15.19	-140.0 to 109.7	No	ns	**0.9860**	B-C
Cribriform vs. Solid	22.65	-121.5 to 166.8	No	ns	**0.9708**	B-D
Cribriform&Tubular vs. Solid	37.84	-87.00 to 162.7	No	ns	**0.8308**	C-D

The statistical data for Fig 6C

Tukey's multiple comparisons test- **CD3**	P value	Mean of Normal	Mean of Tumor	Difference	SE of difference	t ratio	df	q value
No invasion		158.4	62.00	96.40				
Bone invasion		184.0	10.00	174.0				
Perineural invasion	0.020118	185.2	100.1	85.08	27.11	3.138	6.000	0.040639
Bone and Perineural invasion	0.072556	193.9	127.6	66.33	33.05	2.007	10.00	0.073281

Tukey's multiple comparisons test- **CD4**	P value	Mean of Normal	Mean of Tumor	Difference	SE of difference	t ratio	df	q value
No invasion		60.80	20.64	40.16				
Bone invasion		228.0	59.20	168.8				
Perineural invasion	0.652552	75.40	58.75	16.65	35.16	0.4736	6.000	0.659077
Bone and Perineural invasion	0.240531	155.5	69.23	86.24	69.11	1.248	10.00	0.485872

Tukey's multiple comparisons test- **CD8**	P value	Mean of Normal	Mean of Tumor	Difference	SE of difference	t ratio	df	q value	
No invasion			60.80	20.64	40.16				
Bone invasion			228.0	59.20	168.8				
Perineural invasion	No	0.652552	75.40	58.75	16.65	35.16	0.4736	6.000	0.659077
Bone and Perineural invasion	No	0.240531	155.5	69.23	86.24	69.11	1.248	10.00	0.485872

Tukey's multiple comparisons test- **CD19**	P value	Mean of Normal	Mean of Tumor	Difference	SE of difference	t ratio	df	q value
No invasion		269.6	15.20	254.4				
Bone invasion		232.0	1.600	230.4				
Perineural invasion	0.049619	208.2	85.13	123.1	50.18	2.453	6.000	0.025058
Bone and Perineural invasion	0.000123	197.1	4.840	192.3	31.77	6.054	10.00	0.000124
