# Supplementary material for: Transcriptomic insights into adenoid cystic carcinoma via RNA sequencing
Source: Front Genet. 2023 Apr 21;14:1144945. doi: 10.3389/fgene.2023.1144945 (PMC10160386; doi:10.3389/fgene.2023.1144945)
Supplement: Supplementary file 4 [file Table3.DOCX]

Table 3: Gene fusions in adenoid cystic carcinoma

| Sample | FusionName | LeftBreakpoint | RightBreakpoint |
| --- | --- | --- | --- |
| T1 | MYB--NFIB | chr6:135194460:+ | chr9:14088326:- |
|  | MYB--NFIB | chr6:135194451:+ | chr9:14088326:- |
| T2 | SLC12A7--TERT | chr5:1073633:- | chr5:1272280:- |
|  | MYB--NFIB | chr6:135200415:+ | chr9:14088326:- |
| T3 | MYB--NFIB | chr6:135196002:+ | chr9:14102510:- |
|  | MYB--NFIB | chr6:135196002:+ | chr9:14088326:- |
|  | SEPTIN7P2--PSPH | chr7:45768660:- | chr7:56021231:- |
| T4 | MYB--NFIB | chr6:135203324:+ | chr9:14102510:- |
|  | MYB--NFIB | chr6:135203324:+ | chr9:14088326:- |
| T5 | ND |  |  |
| T6 | LHFPL5--CLPSL1 | chr6:35797459:+ | chr6:35786998:+ |
| T7 | ND |  |  |
| T8 | MYBL1--NFIB | chr8:66592440:- | chr9:14088326:- |
|  | KREMEN1--SYN3-AS1 | chr22:29073227:+ | chr22:32583877:+ |
|  | CELSR1--CHADL | chr22:46533627:- | chr22:41229730:- |
|  | SAE1--KIR2DL1 | chr19:47131028:+ | chr19:54775165:+ |
|  | HAS1--VRK3 | chr19:51723925:- | chr19:50016163:- |
|  | EMID1--GMFG | chr22:29206139:+ | chr19:39333126:- |
| T9 | MYBL1--NFIB | chr8:66592440:- | chr9:14088326:- |
|  | ATF2--AC009299.2 | chr2:175093061:- | chr2:161223483:- |
|  | TVP23C--CDRT4 | chr17:15540433:- | chr17:15440285:- |
|  | TONSL-AS1--WDR70 | chr8:144437746:+ | chr5:37516514:+ |
|  | TCP10L--URB1 | chr21:32567941:- | chr21:32385684:- |
| T10 | MYB--NFIB | chr6:135196002:+ | chr9:14102510:- |
| T11 | MYB--NFIB | chr6:135203324:+ | chr9:14102510:- |
|  | ARHGAP24--MAPK10-AS1 | chr4:85995657:+ | chr4:86119984:+ |
| T12 | MYBL1--NFIB | chr8:66592440:- | chr9:14088326:- |
|  | CHST9--AQP4 | chr18:27024116:- | chr18:26862596:- |
|  | AC233976.1--LINC01284 | chrX:51356944:- | chrX:51171871:- |
| T13 | LRRC8B--OOEP | chr1:89525022:+ | chr6:73369385:- |
|  | MYB--NFIB | chr6:135194460:+ | chr9:14102510:- |
|  | NFIB--ARHGEF26-AS1 | chr9:14120440:- | chr3:154090331:- |
|  | MYB--NFIB | chr6:135194451:+ | chr9:14102510:- |
|  | LRRC8B--OOEP | chr1:89549971:+ | chr6:73369385:- |
|  | TAFA2--KRT84 | chr12:62191259:- | chr12:52383798:- |
|  | TAFA2--KRT84 | chr12:62191259:- | chr12:52386990:- |
|  | LRRC8B--OOEP | chr1:89549971:+ | chr6:73394488:- |
| T14 | MYB--NFIB | chr6:135203324:+ | chr9:14102510:- |
|  | MYB--NFIB | chr6:135203324:+ | chr9:14088326:- |
|  | MYB--NFIB | chr6:135203324:+ | chr9:14113081:- |
|  | TVP23C--CDRT4 | chr17:15540433:- | chr17:15440285:- |
|  | TVP23C--CDRT4 | chr17:15545785:- | chr17:15438200:- |
|  | TVP23C--AC005703.3 | chr17:15540433:- | chr17:15416233:- |
| T15 | EYA4--SPTSSB | chr6:133274813:+ | chr3:161359894:- |
|  | PFDN2--ATF3 | chr1:161117952:- | chr1:212615018:+ |
|  | SYNE2--HIF1A | chr14:63853143:+ | chr14:61720382:+ |
|  | CDC14B--PARL | chr9:96619219:- | chr3:183866765:- |
|  | PARL--TTLL11 | chr3:183884722:- | chr9:122039368:- |
|  | DCAF6--ZNF75D | chr1:167937008:+ | chrX:135296039:- |
|  | CDC14B--PARL | chr9:96619219:- | chr3:183844326:- |
|  | ESRRG--LINC02725 | chr1:216564219:- | chr11:128180460:+ |
|  | DAB2IP--SLC18B1 | chr9:121598626:+ | chr6:132774313:- |
|  | TVP23C--CDRT4 | chr17:15540433:- | chr17:15440285:- |
