# Supplementary material for: Transcriptomic insights into adenoid cystic carcinoma via RNA sequencing
Source: Front Genet. 2023 Apr 21;14:1144945. doi: 10.3389/fgene.2023.1144945 (PMC10160386; doi:10.3389/fgene.2023.1144945)
Supplement: Supplementary file 5 [file DataSheet2.DOCX]

Description of the top 20 upregulated and downregulated DE mRNAs

| gene_name | sig |  |
| --- | --- | --- |
| **COL27A1** | up | Encode a member of the fibrillar collagen family, and play a role during the calcification of cartilage and the transition of cartilage to bone. |
| **APBA2** | up | Encode a neuronal adapter protein that interacts with the Alzheimer's disease amyloid precursor protein (APP). It stabilizes APP and inhibits production of proteolytic APP fragments including the A beta peptide that is deposited in the brains of Alzheimer's disease patients. |
| **TUBA1A** | up | Encodes alpha tubulin belong to the tubulin superfamily, which is composed of six distinct families. Mutations in this gene cause lissencephaly type 3 (LIS3) - a neurological condition characterized by microcephaly, intellectual disability, and early-onset epilepsy caused by defective neuronal migration. |
| **TBX1** | up | This gene is a member of a phylogenetically conserved family of genes that share a common DNA-binding domain and plays a major role for this gene in the molecular etiology of DGS/VCFS. |
| **FNDC1** | up | Predicted to act upstream of or within several processes, including cellular response to hypoxia; positive regulation of cardiac muscle cell apoptotic process; and positive regulation of protein phosphorylation. Located in nuclear speck. |
| **PLSCR3** | up | Enables several functions, including calcium-dependent protein binding activity; metal ion binding activity; and phospholipid scramblase activity. Involved in several processes, including cardiolipin biosynthetic process; regulation of apoptotic process; and regulation of release of cytochrome c from mitochondria. Located in cytosol; mitochondrion; and plasma membrane. |
| **FABP7** | up | Encodes a small, highly conserved cytoplasmic protein that bind long-chain fatty acids and other hydrophobic ligands. The encoded protein is important in the establishment of the radial glial fiber in the developing brain. Alternative splicing and promoter usage results in multiple transcript variants encoding different isoforms. |
| **TP53** | up | Encodes a tumor suppressor protein containing transcriptional activation, DNA binding, and oligomerization domains. The encoded protein responds to diverse cellular stresses to regulate expression of target genes, thereby inducing cell cycle arrest, apoptosis, senescence, DNA repair, or changes in metabolism. |
| **ABCC1** | up | Encode a protein which is a member of the superfamily of ATP-binding cassette (ABC) transporters. ABC proteins transport various molecules across extra-and intra-cellular membranes. |
| **EN1** | up | The human engrailed homologs 1 and 2, En1 and En2, encode homeodomain-containing proteins and have been implicated in the control of pattern formation during development of the central nervous system. |
| **MFAP2** | up | Microfibrillar-associated protein 2 is a major antigen of elastin-associated microfibrils and a candidate for involvement in the etiology of inherited connective tissue diseases. |
| **MEX3A** | up | Enables RNA binding activity. Located in P-body and cytosol. |
| **PRAME** | up | This gene encodes an antigen that is preferentially expressed in human melanomas and that is recognized by cytolytic T lymphocytes. It is not expressed in normal tissues, except testis. The encoded protein acts as a repressor of retinoic acid receptor, and likely confers a growth advantage to cancer cells via this function. |
| **STMN1** | up | Encode a ubiquitous cytosolic phosphoprotein proposed to function as an intracellular relay integrating regulatory signals of the cellular environment. The encoded protein is involved in the regulation of the microtubule filament system by destabilizing microtubules. It prevents assembly and promotes disassembly of microtubules. |
| **TRO** | up | Encode a membrane protein that mediates cell adhesion between trophoblastic cells and the epithelial cells of the endometrium. The encoded protein participates in cell signalling during embryo implantation, and may also be involved in cancer formation. This gene is located near several other closely related genes on chromosome X. |
| **OBP2B** | up | Predicted to enable small molecule binding activity. Predicted to be involved in chemosensory behavior. Predicted to be located in extracellular region. Predicted to be active in extracellular space. |
| **ITGA9** | up | Encode an alpha integrin. The protein forms an integrin that is a receptor for VCAM1, cytotactin and osteopontin when bound to the beta 1 chain. |
| **TTYH1** | up | Encode a member of the tweety family of proteins. Members of this family function as chloride anion channels. The encoded protein functions as a calcium(2+)-independent, volume-sensitive large conductance chloride(-) channel. Three transcript variants encoding distinct isoforms have been identified for this gene. |
| **SOX4** | up | This intronless gene encodes a member of the SOX (SRY-related HMG-box) family of transcription factors involved in the regulation of embryonic development and in the determination of the cell fate. The encoded protein may act as a transcriptional regulator after forming a protein complex with other proteins, such as syndecan binding protein (syntenin). The protein may function in the apoptosis pathway leading to cell death as well as to tumorigenesis and may mediate downstream effects of parathyroid hormone (PTH) and PTH-related protein (PTHrP) in bone development. |
| **CASC15** | up | This gene produces a long non-coding RNA that may regulate cell proliferation. This RNA is upregulated in hepatocellular carcinoma, where it is thought to function as an oncogene. However, some splice variants of this gene may function as a tumor suppressor in neuroblastoma and other tumor types. Circular RNA variants were observed at this gene. |
| **CST2** | down | This gene is located in the cystatin locus and encodes a secreted thiol protease inhibitor found at high levels in saliva, tears and seminal plasma. |
| **SMR3B** | down | Predicted to enable endopeptidase inhibitor activity. Predicted to be involved in cellular response to lipopolysaccharide; negative regulation of peptidase activity; and regulation of sensory perception of pain. Located in extracellular exosome. |
| **KLK1** | down | This protein is functionally conserved in its capacity to release the vasoactive peptide, Lys-bradykinin, from low molecular weight kininogen. |
| **CTBS** | down | Chitobiase is a lysosomal glycosidase involved in degradation of asparagine-linked oligosaccharides on glycoproteins |
| **DHRS2** | down | Encode a member of the short-chain dehydrogenases/reductases (SDR) family, which has over 46,000 members. Members of this family are enzymes that metabolize many different compounds, such as steroid hormones, prostaglandins, retinoids, lipids and xenobiotics. |
| **STATH** | down | Predicted to be involved in ossification. |
| **SH3BGRL2** | down | Predicted to enable SH3 domain binding activity. Located in nuclear membrane and nucleoplasm. |
| **HTN1** | down | Encode a member of the histatin family of small, histidine-rich, cationic proteins. They function as antimicrobial peptides and are important components of the innate immune system. Histatins are found in saliva and exhibit antibacterial, antifungal activities and function in wound healing. |
| **SLC13A5** | down | Encode a protein belonging to the solute carrier family 13 group of proteins. This family member is a sodium-dependent citrate cotransporter that may regulate metabolic processes. Mutations in this gene cause early infantile epileptic encephalopathy 25. |
| **SLC9A1** | down | Encode a Na+/H+ antiporter that is a member of the solute carrier family 9. The encoded protein is a plasma membrane transporter that is expressed in the kidney and intestine. This protein plays a central role in regulating pH homeostasis, cell migration and cell volume. This protein may also be involved in tumor growth. |
| **HTN3** | down | Encode a member of the histatin family of small, histidine-rich, cationic proteins. They function as antimicrobial peptides and are important components of the innate immune system. Histatins are found in saliva and exhibit antibacterial, antifungal activities and function in wound healing. |
| **PON3** | down | This gene is a member of the paraoxonase family and lies in a cluster on chromosome 7 with the other two family members. The encoded protein is secreted into the bloodstream and associates with high-density lipoprotein (HDL). The protein also rapidly hydrolyzes lactones and can inhibit the oxidation of low-density lipoprotein (LDL), a function that is believed to slow the initiation and progression of atherosclerosis. |
| **FXYD2** | down | This gene encodes a member of the FXYD family of transmembrane proteins. This particular protein encodes the sodium/potassium-transporting ATPase subunit gamma. Mutations in this gene have been associated with Renal Hypomagnesemia-2. |
| **WWC1** | down | The protein encoded by this gene is a cytoplasmic phosphoprotein that interacts with PRKC-zeta and dynein light chain-1. Alleles of this gene have been found that enhance memory in some individuals. Three transcript variants encoding different isoforms have been found for this gene. |
| **PDCD4** | down | This gene is a tumor suppressor and encodes a protein that binds to the eukaryotic translation initiation factor 4A1 and inhibits its function by preventing RNA binding. |
| **CTPS1** | down | Encode an enzyme responsible for the catalytic conversion of UTP (uridine triphosphate) to CTP (cytidine triphospate). This reaction is an important step in the biosynthesis of phospholipids and nucleic acids. Activity of this proten is important in the immune system, and loss of function of this gene has been associated with immunodeficiency. |
| **FUT8** | down | Encode an enzyme belonging to the family of fucosyltransferases. The product of this gene catalyzes the transfer of fucose from GDP-fucose to N-linked type complex glycopeptides. This enzyme is distinct from other fucosyltransferases which catalyze alpha1-2, alpha1-3, and alpha1-4 fucose addition. The expression of this gene may contribute to the malignancy of cancer cells and to their invasive and metastatic capabilities. |
| **ATP2B2** | down | The protein encoded by this gene belongs to the family of P-type primary ion transport ATPases characterized by the formation of an aspartyl phosphate intermediate during the reaction cycle. These enzymes remove bivalent calcium ions from eukaryotic cells against very large concentration gradients and play a critical role in intracellular calcium homeostasis. The mammalian plasma membrane calcium ATPase isoforms are encoded by at least four separate genes and the diversity of these enzymes is further increased by alternative splicing of transcripts. The expression of different isoforms and splice variants is regulated in a developmental, tissue- and cell type-specific manner, suggesting that these pumps are functionally adapted to the physiological needs of particular cells and tissues. This gene encodes the plasma membrane calcium ATPase isoform 2. |
| **BLM** | down | BLM (BLM RecQ Like Helicase) is a Protein Coding gene. Diseases associated with BLM include Bloom Syndrome. |
| **PRR27** | down | Located in extracellular exosome. is a Protein Coding gene. Diseases associated with PRR27 include Cerebellar Ataxia, Cayman Type and Hereditary Wilms' Tumor. |

The above information is sourced from https://www.genecards.org/.
