# Supplementary material for: Transcriptomic insights into adenoid cystic carcinoma via RNA sequencing
Source: Front Genet. 2023 Apr 21;14:1144945. doi: 10.3389/fgene.2023.1144945 (PMC10160386; doi:10.3389/fgene.2023.1144945)
Supplement: Supplementary file 6 [file Table4.DOCX]

Table 4: Different types of gene fusions

|  |  | LeftBreakpoint | RightBreakpoint |
| --- | --- | --- | --- |
| MYB—NFIB | T1 | chr6:135194460:+ | chr9:14088326:- |
|  |  | chr6:135194451:+ | chr9:14088326:- |
|  | T2 | chr6:135200415:+ | chr9:14088326:- |
|  | T3 | chr6:135196002:+ | chr9:14102510:- |
|  |  | chr6:135196002:+ | chr9:14088326:- |
|  | T4 | chr6:135203324:+ | chr9:14102510:- |
|  |  | chr6:135203324:+ | chr9:14088326:- |
|  | T10 | chr6:135196002:+ | chr9:14102510:- |
|  | T11 | chr6:135203324:+ | chr9:14102510:- |
|  | T13 | chr6:135194460:+ | chr9:14102510:- |
|  |  | chr6:135194451:+ | chr9:14102510:- |
|  | T14 | chr6:135203324:+ | chr9:14102510:- |
|  |  | chr6:135203324:+ | chr9:14088326:- |
|  |  | chr6:135203324:+ | chr9:14113081:- |
| MYBL1--NFIB | T8 | chr8:66592440:- | chr9:14088326:- |
|  | T9 | chr8:66592440:- | chr9:14088326:- |
|  | T12 | chr8:66592440:- | chr9:14088326:- |
| TVP23C—CDRT4 | T9 | chr17:15540433:- | chr17:15440285:- |
|  | T14 | chr17:15540433:- | chr17:15440285:- |
|  |  | chr17:15545785:- | chr17:15438200:- |
|  | T15 | chr17:15540433:- | chr17:15440285:- |
